# Shotgun metagenomics on indoor air for surveillance of respiratory, enteric, and skin viruses in a Belgian daycare setting, January to December 2022

**DOI:** 10.2807/1560-7917.ES.2025.30.38.2400711

**Published:** 2025-09-25

**Authors:** Mustafa Karatas, Caspar Geenen, Els Keyaerts, Lore Budts, Joren Raymenants, Charlotte Eggers, Bastiaan Craessaerts, Emmanuel André, Jelle Matthijnssens

**Affiliations:** 1 Laboratory of Viral Metagenomics, Clinical and Epidemiological Virology, Rega Institute, Department of Microbiology, Immunology and Transplantation, KU Leuven, Leuven, Belgium; 2 Laboratory of Clinical Microbiology, KU Leuven, Leuven, Belgium; 3 Department of Laboratory Medicine, National Reference Center of Respiratory Pathogens, University Hospitals Leuven, Leuven, Belgium; * These authors contributed equally.

**Keywords:** indoor air surveillance, viral metagenomics, environmental surveillance

## Abstract

**BACKGROUND:**

Hospital-based communicable disease surveillance may be costly during large outbreaks and often misses mild or asymptomatic infections. It can be enhanced by environmental surveillance, which monitors circulating pathogens, even from asymptomatic carriers.

**AIM:**

We investigated if tracking viruses in indoor air could be used for their surveillance in a community setting. We also tested the value of untargeted metagenomics to identify viruses in air samples.

**METHODS:**

Weekly indoor air samples were collected with active air samplers from January until December 2022 from a daycare centre in Leuven, Belgium. Samples were analysed using respiratory and enteric quantitative (q)PCR panels, as well as with untargeted metagenomics, enabling both targeted and agnostic viral detections.

**RESULTS:**

Human-associated viruses were detected in 40 of 42 samples across the study period, with MW polyomavirus being most prevalent (33 samples). Respiratory agents such as rhinoviruses and RSV-B and enteric viruses including rotavirus A, astrovirus, and adenovirus appeared at epidemiologically expected times. Skin-associated viruses were also observed, notably Merkel cell polyomavirus and STL polyomavirus. Metagenomics enabled reconstructing multiple complete genomes, distinguishing viral subtypes and detecting copresence of closely related variants. Additionally, several animal, insect, fungal, and plant viruses were found, reflecting both indoor and outdoor environmental exposure.

**CONCLUSION:**

Indoor air monitoring, combined with untargeted metagenomics, demonstrates a potential to support virus surveillance. This approach can allow monitoring circulation of viruses in community settings, including those causing asymptomatic or mild infections. By enabling to reconstruct complete viral genomes, it allows detailed variant tracking, facilitating adapted public health responses.

**Key public health message**

**What did you want to address in this study and why?**

Hospital diagnostics are vital for patient care but limited for timely surveillance: they miss mild or asymptomatic infections, may detect viruses only after community spread, and overlook pathogens not routinely tested. We investigated whether indoor-air monitoring can reveal viruses circulating in the community.

**What have you learnt from this study?**

In 2022, air samples from a daycare centre were tested for genetic material of either (i) specific viruses with qPCR techniques or (ii) of any virus with a metagenomics method. In 40 of 42 total samples collected during the year, human viruses were detected, despite children at the daycare appearing healthy. Tracking skin, respiratory, or enteric viruses through indoor air sampling was feasible. Known and new viruses could be simultaneously detected.

**What are the implications of your findings for public health?**

Indoor air monitoring can complement current virus surveillance systems by detecting known and novel viruses. Tracking viruses in the community before detections in hospitals occur, may allow health officials to more timely prevent their circulation among people, gain more information on their epidemiology and improve outbreak responses.

## Introduction

Conventionally, infectious disease surveillance has revolved around patient-centred approaches, focusing on specific diseases or syndromes (e.g. respiratory, enteric symptoms) using methods such as pathogen culturing and molecular techniques (PCR or quantitative (q)PCR panels) on individual patient samples [1]. While individual diagnostics can be valuable for immediate patient care or to assess the extent of an outbreak, these methods are often underused for broader surveillance due to cost, invasiveness and failure to detect less common or novel viruses [2]. Moreover, due to their focus on illness, they tend to miss mild or asymptomatic infections. Therefore, environmental surveillance has emerged as a relevant complement to individual testing for tracking pathogens in larger populations, allowing to monitor viral circulation in communities without relying on patient sampling.

Since the 2010s, environmental surveillance for pathogens in media such as wastewater, soil, rivers, and indoor air, has attracted growing interest, with wastewater being the most widely used method [3-5]. Wastewater analysis has effectively complemented other surveillance systems by tracking severe acute respiratory syndrome coronavirus 2 (SARS-CoV-2), the variants of concern of this virus, and other respiratory and enteric pathogens using targeted PCR methods [6-8]. However, wastewater surveillance has limitations, including reduced precision in pinpointing specific locations or population subgroups, potential delays or signal dilution due to rainfall, and dependency on water usage [9]. As an alternative approach, indoor air sampling can be performed using active or passive samplers, with sampling conducted at specific locations for precise surveillance purposes. Active air sampling facilitates immediate sample collection within a short time frame and offers higher sensitivity, as demonstrated especially in studies on SARS-CoV-2 [10]. In 2023 and 2024, our teams and others have demonstrated the potential of active indoor air sampling for monitoring viruses [11-14]. This method has successfully identified a variety of viruses, including respiratory and enteric viruses (e.g. respiratory syncytial virus (RSV), influenza C, human parainfluenza virus) in community settings.

The methodologies employed to detect pathogens in environmental surveillance are usually similar to those employed in clinical diagnostics [1,15], frequently using molecular based single- or multi-targeted (qPCR or multiplex qPCR) tests. Targeted test methods tend to have high sensitivity and specificity. Previously, some studies have focused on the detection of viruses and bacteria with targeted methods from indoor air of various community settings [11,13,14], revealing temporal trends of virus circulation among specific populations and highlighting the early warning potential of indoor air sampling in real-world situations. However, for more precise genetic identification or subtyping, additional techniques such as Sanger sequencing or whole genome sequencing (WGS) are often necessary. Characterising novel or variant viruses can be challenging with both methods, because they require specific genetic sequence knowledge of viruses of interest.

Next generation sequencing (NGS)-based 'untargeted' shotgun metagenomic methods can overcome this limitation. These methods often use a combination of viral enrichment, followed by deep sequencing using various NGS technologies and appropriate bioinformatic tools [16]. An established method, Novel Enrichment Techniques of Viromes (NetoVIR) [17], has proven effective for viral enrichment across various sample types, including faecal [18,19], insect [20,21], and plant samples [22], facilitating virome analysis with minimal bias and the discovery of novel viruses. This approach allows to identify pathogens and provide genomic insights as a 'pathogen-agnostic' tool [23]. For indoor air surveillance, NGS faces challenges such as low biomass and contaminating nucleic acid, despite its relatively cleaner nature compared with soil, wastewater or stool [24]. Nevertheless, Minor et al*.* used RNA virus-targeted NGS to analyse 20 samples from various indoor community settings. They detected not only respiratory viruses such as influenza A and C, but also rotaviruses and astrovirus, which are transmitted through the faecal–oral route [25].

In this study, air samples spanning a full year at a daycare centre were subjected to qPCR and untargeted metagenomics, to investigate the viruses they contained and better understand the indoor air virome and its dynamics. In addition, our objective was to explore the potential for viral surveillance of air sampling combined with metagenomics with reference-guided and *de novo* bioinformatics approaches.

## Methods

### Setting and sample collection 

Between January 2022 and December 2022, 90 indoor air samples were collected from a daycare centre in Leuven, hosting children aged 0 to 3 years old (with on an average day, 15 children and 3 adults aged ≥ 18 years old occupying the establishment). 

We aimed to have from each month at least four samples (collected on a weekly basis), to explore different methods of virus detection and genetic analysis. However, after using parts of each sample for testing with the respiratory qPCR panel and initial optimisation of our assays, sufficient material was left to conduct metagenomic and enteric qPCR panel testing on 42 samples.

Sampling was done for 2 hours between 9.00 and 11.00 am in the changing room of the daycare centre which has the air extraction vents, and which is connected by two open doors to the main area where children are present. When the daycare centre was closed (e.g. holidays), no samples were collected. 

For sampling, an AerosolSense active air sampler was used [11]. Ambient air was sampled with a rate of 200 L/min through a vertical collection pipe and impacted onto the AerosolSense Capture Media (Thermo Fisher Scientific, Waltham, Massachusetts (MA)). This method captures particles suspended in the air — which may include resuspended surface dust — while avoiding particles following a semi-ballistic trajectory.

Concentrations of carbon dioxide (CO_2_) were measured using a remote climate sensor (Elsys ERC CO2, Umeå, Sweden; placed next to the air sampler). Natural ventilation was also estimated by a Likert scale (no natural ventilation, one window open, door open, multiple windows open, door and window open).

### Sample processing

After removing the standard cartridges from the sampler, samples were transported to the laboratory on the same day that they were collected. Samples were dissolved in 2 mL of universal transport medium (UTM), to be used for respiratory and enteric multiplex qPCR panels as well as metagenomics (see below). Samples were stored at 4 °C until processing with the respiratory multiplex qPCR panel, which was conducted, whenever possible, on the same day as the sample collection. If storage was required over the weekend, samples were frozen at − 80 °C. For the respiratory panel, 200 µL of each sample was used. The remaining volume was immediately stored at − 20 °C until proceeding with metagenomics and the enteric qPCR panel, which occurred in February 2023, on samples which had undergone two or less freeze−thaw cycles. As negative controls, UTM (used to dissolve samples) was processed in parallel and sequenced as deep as samples.

### Respiratory and enteric multiplex qPCR panels

Samples dissolved in UTM were subjected to multiplex qPCR testing. The detailed protocols for these procedures have been previously described [11,26]. The respiratory panel is designed to detect 22 viruses and seven bacterial pathogens and was applied on 90 samples when they were collected. In addition, the enteric panel detects 17 non-viral pathogens (4 parasites and 13 bacteria) and six viruses, specifically human adenovirus F40/F41, norovirus GI, norovirus GII, rotavirus A, astrovirus, and sapovirus (genogroups GI, GII, GIV, and GV). The enteric panel was applied to the same 42 samples used for shotgun metagenomics (see below).

### Shotgun metagenomics

Samples were subjected to NetoVIR [27]. They were homogenised by vortexing for 10 seconds and homogenous samples were centrifuged at 17,000 g for 3 min and filtered with a 0.8 µm polyethersulfone (PES) centrifugal filter to eliminate eukaryotic cells and bacteria. Free floating nucleic acids were removed by benzonase and micrococcal nuclease while being incubated for 2 hours at 37 °C. A modified Whole Transcriptome Amplification 2 (WTA2) kit was used to amplify both DNA and RNA. To obtain DNA amplification, the initial denaturation step was performed at 95 °C instead of 70 °C. Library preparation was done using a Nextera XT DNA Sample Preparation kit and sequencing was performed on a NovaSeq platform (Illumina).

### Clinical data collection

At the daycare centre — where samples were collected — parents were advised to keep their children at home if they exhibited any symptoms of illness such as fever, runny nose, or diarrhoea. This led to our study population consisting of seemingly ‘healthy’ or asymptomatic individuals.

We collected the results of respiratory panel tests for enterovirus (including rhinovirus) performed at the clinical laboratory of University Hospitals Leuven during the same period as the air sample collection. The PCR targets also included non-rhinovirus enteroviruses. Clinical respiratory qPCR panels are performed according to specific clinical indications. In immunocompetent individuals, these panels are used for respiratory infections requiring intensive care admission or those unresponsive to initial therapy for a respiratory infection. For immunocompromised patients, respiratory panels are more commonly used when lower respiratory tract infections are suspected.

For rotavirus A infections in the age group of 0 to 3 years old, we used data from the national reference centre (NRC) for rotavirus in Leuven, Belgium in 2022. Samples of the NRC were collected through the nationwide laboratory system on a voluntary basis and under the regulations defined by the Royal Decree of 09/02/2011 [28].

### Bioinformatics analyses

We employed reference-guided assembly and *de novo* assembly on the reads obtained from sequencing. Analyses were conducted on the Flemish Supercomputer Center ‘Vlaams Supercomputer Centrum’ (VSC) KU Leuven servers.

#### Reference-guided assembly

To identify human and animal-associated viruses, we used EsViritu [29] (v0.2.3) and nucleotide (nt) basic local alignment tool (BLASTN). EsViritu employs a database of all human and animal viruses in GenBank up to 16 November 2022 (EsViritu DB v2.0.2), curated to include one representative genome per 95% average nt identity cluster. Reads underwent quality control, adapter trimming, and mapping to reference sequences with 90% identity and 90% coverage. Viruses were considered present if the consensus sequence exceeded 200 bp or 10% of the reference genome and at least two reads mapped to it. Consensus sequences were obtained using Samtools consensus [30] and subsequently verified using BLASTN. Reference-guided assembly only showed human papillomavirus (HPV)-21 with 39 reads in one negative control, which resulted in a 236-nt long consensus sequence, found to be identical to that from a higher viral load sample with the same virus, and was therefore classified as cross-contamination from this sample.

#### *De novo* assembly

For *de novo* assembly of viral genomes (or contigs), we used the Virome Paired-End Reads pipeline (ViPER) [31]. The pipeline processed quality-controlled and adapter-trimmed reads, performing *de novo* triple-assembly using metaSPAdes [32]. Contigs were then clustered at 95% identity and 85% coverage to reduce redundancy, first within individual samples and then among all samples. Reads were mapped back to contigs > 1 kbp, considering those with over 50% horizontal coverage present in each sample. Contig identification was subsequently performed using DIAMOND [33] for eukaryotic viruses, geNomad [34] for bacteriophages, and BLASTN for identification of genetic material associated to other entities (i.e. fungi, plant, human). For *de novo* assembly, all contigs present in negative controls were treated as contaminants and removed before downstream analyses.

### Statistical analyses

Statistical analyses for the difference of detections in the daycare’s open and closed window periods of non-human infecting and human-infecting viruses were conducted using the chi-square test and performed in RStudio. The analysis assessed the association between seasonality and the presence of these viruses. A p value of less than 0.05 was considered statistically significant.

## Results

On average, 25 million reads were produced per sample. The findings based on their analyses by both EsViritu (thereafter referred to as ‘reference-guided assembly’), and ViPER, (referred to as ‘*de novo* assembly’) are further described together unless otherwise specified.

### Respiratory, enteric and skin viruses revealed by untargeted shotgun metagenomics in indoor air

**Figure 1:**
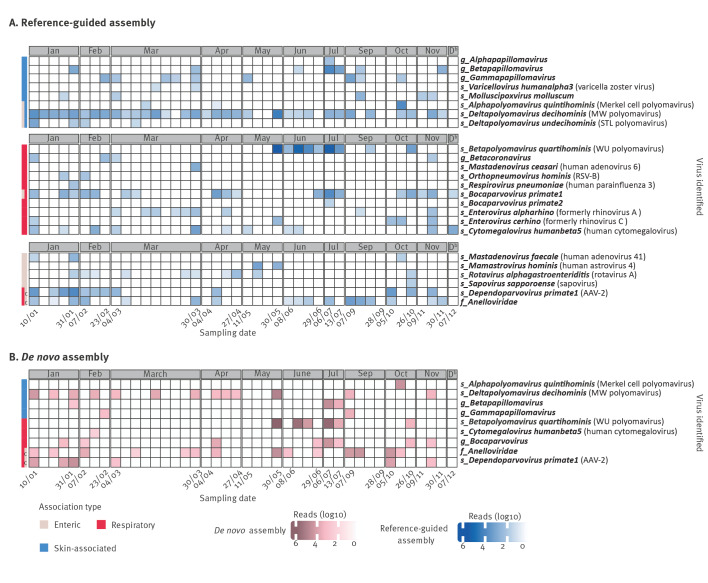
Human-associated viruses identified in indoor air samples of a daycare centre via shotgun metagenomics with (A) reference-guided and (B) *de novo* assembly, Leuven, Belgium, January–December 2022^a^ (n = 42 air samples shown)

AAV-2: adeno-associated virus 2; f: family; g: genus; s: species.

^a^ The daycare centre was closed between mid-July and September, so no samples were collected during this period. Dates with no sampling including all the month of August 2022 are omitted from the graph.

^b^ D: December.

^c^ AAV-2 is known to infect a wide range of human cells and anelloviruses do not have a clear host cell, however both are reported to shed from the enteric and respiratory tracts.

Using reference-guided assembly, we identified human-associated viruses in 40 of 42 samples; the list of viruses identified in each sample with read counts can be found in the Supplementary information 1. *De novo* assembly identified human-associated viruses in 29 of 42 samples (Figure 1). Several respiratory viruses were detected during the study period, including human bocavirus 1, 2 and 3 (species *Bocaparvovirus primate1–2,* number of detections (*n_dt_*) = 18), *Enterovirus alpharhino* and *Enterovirus cerhino* (formerly *Rhinovirus* *A* (*n_dt_* = 9) and *C* (*n_dt_* = 5)), human adenovirus 6 (*n_dt_* = 1), viruses belonging to the genus *Betacoronavirus* (human coronavirus HKU1, *n_dt_* = 3 and OC43, *n_dt_* = 1) and RSV-B (*n_dt_* = 2). WU polyomavirus was consistently detected from the end of May until the summer holiday closure in July (*n_dt_* = 7 during that period), and it accounted for the highest total number of human-infecting virus reads in our study. Among the identified respiratory viruses, we obtained complete genomes of WU polyomavirus and human bocavirus 1.

On the X-axis, the first and last sample collection in each month is labelled.

Enteric virus reads detected in the air included rotavirus A (*n_dt_* = 10), human astrovirus 4 (*n_dt_* = 2), sapovirus (*n_dt_* = 1), and human adenovirus 41 (*n_dt_* = 3). The highest horizontal genome coverage from these strictly enteric pathogens was reached for human astrovirus 4 with more than 70% coverage. While these pathogens are associated with gastroenteritis or respiratory disease in young children [35,36], other viruses identified in the current study, such as MW polyomavirus [37], STL polyomavirus [38,39], human bocavirus 1–3 [40,41], anelloviruses [42], and adeno-associated virus-2 (AAV-2) [43] are described in stool without being associated with disease. Anelloviruses were detected in 22 samples, and AAV-2 was identified in 12 samples. In five of the 22 samples testing positive for anelloviruses, these viruses were only identified through *de novo* assembly, highlighting their high genetic variability, which may have complicated detection by reference-based approaches.

Notably, the most frequently identified virus was MW polyomavirus, present in 33 of the 42 samples collected throughout the year. This virus showed 98.8% nt similarity to a strain reported to be shedding from the skin of healthy individuals in the United States (US) [44]. Several skin-associated polyomaviruses were detected, including STL polyomavirus and Merkel cell polyomavirus [45]. Additionally, varicella zoster virus (VZV) and variety of HPVs (18 different types, including HPV-151, HPV-120, HPV-129, HPV-9, and HPV-76, detected in 14 samples) were identified.

### Seasonality of respiratory viruses was reflected in the air

Examination of the seasonality of respiratory viruses with shotgun metagenomics revealed distinct patterns. Rhinoviruses were detected throughout the year, showing no clear seasonal pattern. Bocaparvoviruses were identified frequently in blocks of several consecutive positive samples (mid-January, April, end-June, mid-October), presumably representing outbreaks. In contrast, viruses with well-established seasonal patterns, such as human coronaviruses and RSV, typically peak during the winter months [46,47] and were only detected in the months of January, February, and November in our study (Figure 1A).

To increase the resolution for seasonality analyses, we used qPCR respiratory panel results from 90 samples (the same 42 samples as above, plus 48 additional indoor air samples from the same year and location, see methods). RSV was detected only from January to April (*n_dt_* = 12, quantification cycle (Cq) range: 32.0–39.1) and November to December (*n_dt_* = 7, Cq range: 32.9–35.3). Similarly, human parainfluenza virus 3 was found from January to March (*n_dt_* = 12, Cq range: 33.5–38.7) and once in May (*n_dt_* = 1, Cq: 35.3). Human coronaviruses (HKU-1 and OC43) were detected 42 times, mostly between January and March (*n_dt_* = 31, Cq range: 27.9–37.8) and in November to December (*n_dt_* = 6, Cq range: 27.9–37.8). Furthermore, the respiratory qPCR panel and shotgun metagenomics did not detect any SARS-CoV-2, whereas a more sensitive TaqPath assay detected SARS-CoV-2 in 45 of 90 samples; qPCR results for respective viruses can be found in Supplementary information 2. S1.

### Indoor air surveillance as a non-invasive way to follow temporal trends of rhinovirus and rotavirus A

A recent study revealed that the number of infant hospitalisations due to human rhinoviruses represent up to two-thirds of those due to RSV [48], while rotavirus remains the most common gastroenteritis cause in children younger than 5 years old worldwide. In our data RSV was only identified in two indoor samples using shotgun metagenomics, possibly because infants with more severe disease were kept away from the daycare centre. On the other hand, rotavirus and rhinoviruses were consistently detected (Figure 1A), and therefore, they were selected for a more thorough comparison to other openly available surveillance data.

**Figure 2:**
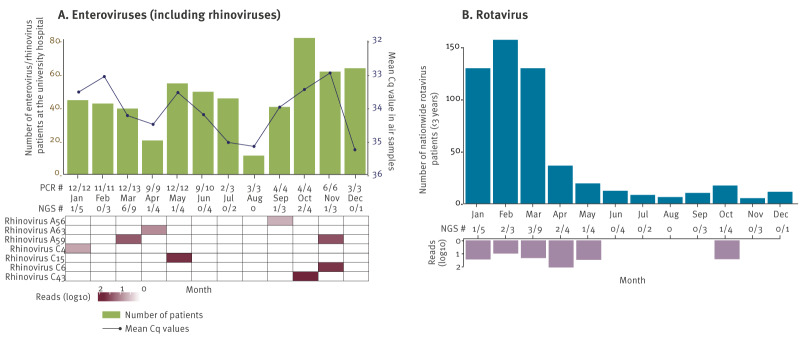
Monthly numbers of patients testing (A) enterovirus positive at the University Hospitals Leuven laboratory and (B) rotavirus positive at the NRC together with detection data for these respective viruses in a daycare centre air samples in Leuven, Belgium, 2022

For rhinovirus epidemiology, data from patients of all age with respiratory symptoms from the University Hospitals Leuven, who tested positive for enteroviruses (which includes rhinoviruses; see methods) using the respiratory qPCR panel, were used as a proxy (Figure 2A, brown bars). The same qPCR panel was applied to all indoor air samples (n = 90), revealing a 96.7% positivity rate (87/90) (indicated with ‘PCR #’ in Figure 2A). Shotgun metagenomics identified rhinoviruses in 13 of the 42 samples and not every month, which is a lower positivity rate compared with qPCR panel tests. The inverse average Cq qPCR values per month showed similar trend to clinical cases between January–March, May–July and September–November. Despite low read counts (read counts can be found in Supplementary Information 1), our shotgun metagenomics data allowed identification and subtyping of rhinoviruses (Figure 2A, heatmap).

Cq: quantification cycle; NGS: next generation sequencing; NRC: national reference centre; qPCR: quantitative PCR.

A. Chart with monthly enterovirus positive (including rhinoviruses) patients of all ages at the University Hospitals Leuven laboratory (green bars) and average qPCR Cq values per month (black line) measured from indoor air samples collected at the daycare centre between January–December 2022. The qPCR positivity rates are displayed on the X axis and below these the month of testing. Below the chart, a heatmap created using data from shotgun metagenomics of the daycare air is presented. Above the heatmap, the positivity rates of the NGS samples are presented for each month. B. Chart with monthly rotavirus cases under 3 years old in Belgium in 2022 coupled with an inverted bar plot of aligned reads of rotavirus A (log10 scale) per month, obtained from our analyses. The monthly positivity rates obtained by NGS are displayed on the X axis of the inverted bar plot.

To compare indoor air monitoring data with actual rotavirus A circulation measured by surveillance, nationwide data from the NRC for rotavirus A (see methods) among children younger than 3 year*s* old were used (Figure 2B). Unlike rhinoviruses, rotavirus A was not identified in daycare air using the qPCR enteric panel (0/42), but it was detected in 10 samples (January to May and October) using untargeted shotgun metagenomics. These detections corresponded well with nationwide data and the seasonality reported by the NRC for rotaviruses in Belgium (Figure 2B).

### Untargeted shotgun metagenomics allows detailed phylogenetic analyses, and discrimination of distinct viral variants

**Figure 3:**
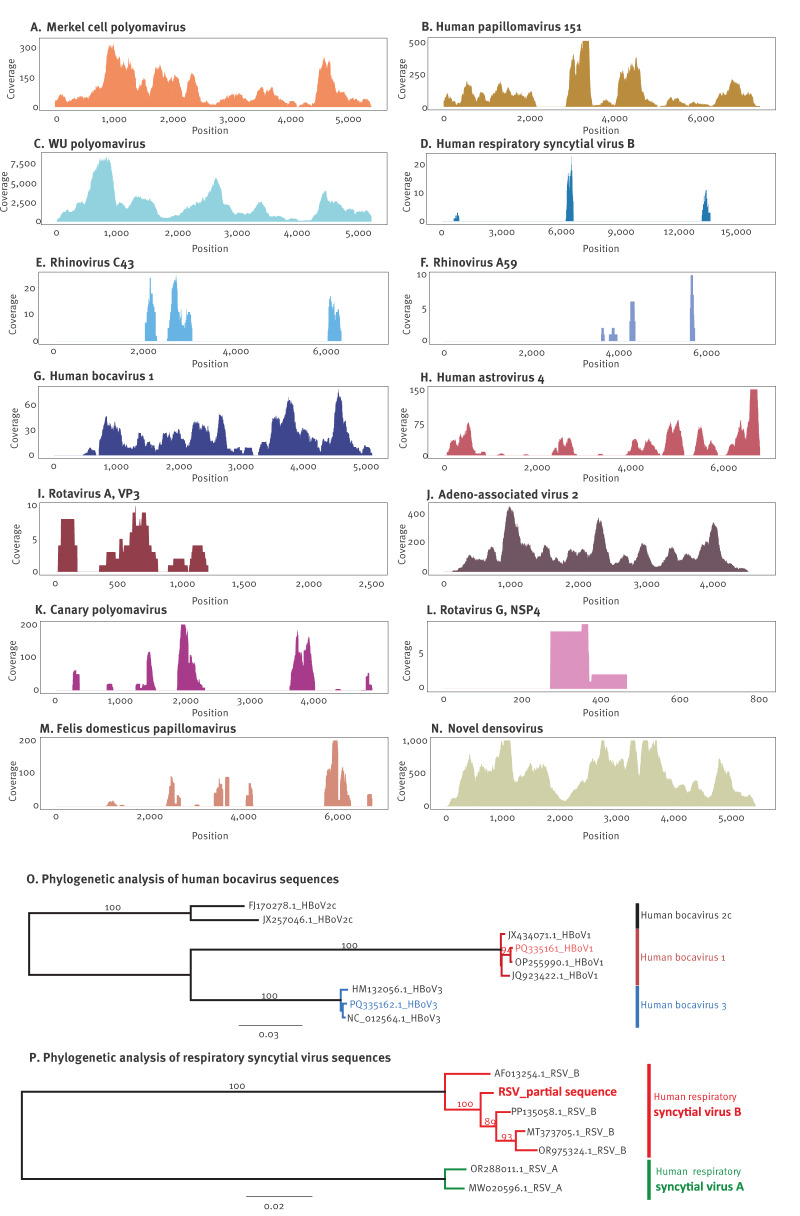
(A–N) Sequencing coverage plots of genetic sequences of viruses detected in indoor air samples of a daycare and phylogenetic analyses of sequences of (O) two human bocaviruses and (P) one RSV identified in some samples, Belgium, January–December 2022

HBoV: human bocavirus; NSP4: non-structural protein 4; RSV: respiratory syncytial virus.

In addition to the detection (absence or presence) of a particular pathogen, further molecular characterisation of viral genomes, such as subtyping or identifying mutations responsible for resistance against therapies can be important for public health purposes. Therefore, we aimed to further leverage the obtained NGS data for the molecular characteristics of viral genomes found in indoor air samples. The data allowed us to reconstruct several partial and (near) complete genomes of several skin-associated (Figure 3A–C) respiratory (Figure 3D–F) and enteric viruses (Figure 3G–I) as well as multiple near-complete genomes of AAV-2 (Figure 3J). Moreover, various animal (bird and cat) viruses (Figure 3K–M) and a complete genome of a novel densovirus were identified (Figure 3N). These obtained genomic data can further be used for phylogenetic analysis, as exemplified for the partial RSV genome data and the near-complete genomes of bocaviruses to reliably study their taxonomy and evolutionary relationships (Figure 3O–P). The partial genome of RSV clustered closely with RSV-B sequences, whereas bocavirus genomes showed over 99% similarity to human bocavirus 1 (OP255990) and human bocavirus 3 (NC_012564), reference sequences. Further examples of phylogenetic analyses for AAV-2, WU and MW polyomaviruses, HPV-151, and the partial genomes of felis domesticus papillomavirus, a novel densovirus, a divergent canary polyomavirus, and human astrovirus 4, are provided in Supplementary information 2 S2.

A–N. Coverage plots of viruses found in indoor air samples. The Y-axis for each panel is different and shows the depth of coverage, while the X-axis shows nt positions. O–P. Phylogenetic trees of two human bocaviruses and RSV found in indoor air samples. Trees are constructed by the maximum likelihood method and numbers above the branch points denote the confidence level of the relationship of the paired sequences determined by bootstraps (n = 100). Branch lengths are measured in substitutions per site (e.g. the 0.02 scale bar indicates ~2% sequence divergence).

Interestingly, we detected WU polyomavirus in the daycare centre for the first time at the end of May, and it was consistently present for nearly 2 months with high abundance, until the start of the summer break (Figure 1A). This prompted us to investigate whether this prolonged detection was due to viral shedding of a single strain by one or more individuals or if there were multiple introductions of distinct viruses. Therefore, we reconstructed four near-complete genomes of WU polyomavirus from four samples collected between the end of May and July (30 May, 15 June, 22 June and 6 July). We show that the viral genomes collected at the first two time points are identical, whereas the third sample showed 12 mutations, suggesting the introduction of a second WU polyomavirus variant into the daycare centre (mutations in different variants and read level analysis can be found in Supplementary information 2. S3). Finally, our in-depth read-level analysis suggests that both of these variants were present in the fourth sample.

### Untargeted shotgun metagenomics on indoor air samples can reflect temporal dynamics of both the indoor and outdoor environment

**Figure 4:**
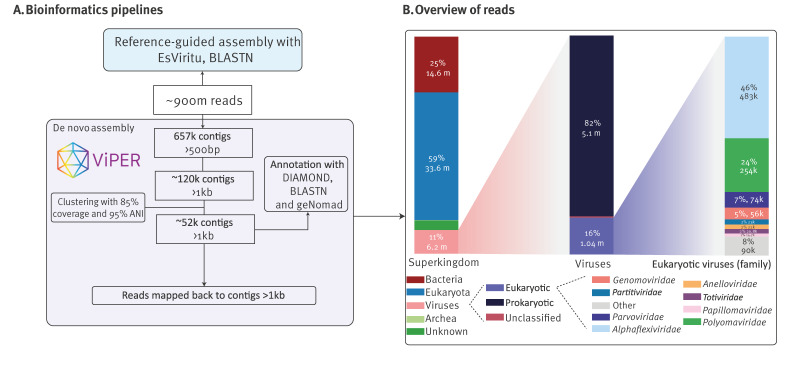
(A) Bioinformatics pipelines for identifying viruses by untargeted metagenomics in indoor air samples of a daycare centre and (B) overview of obtained reads mapped back to contigs, Leuven, Belgium, January–December 2022

ANI: average nucleotide identity; BLASTN: nucleotide (nt) basic local alignment tool; VIPER: virome paired-end reads pipeline.

Next, we focused on the air virome linked to other entities such as plants, animals (including insects), and fungi. Although contigs derived from bacteriophages (prokaryotic viruses) were also abundantly identified, due to their very short average contig length (ca 1kb), we did not further explore them (Figure 4).

**Figure 5:**
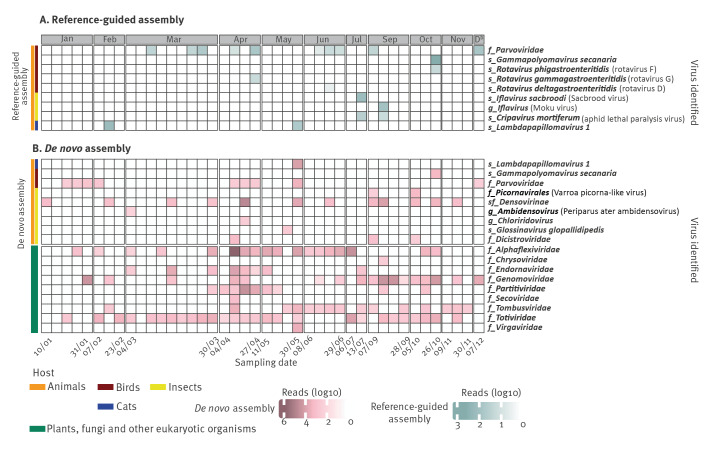
Detections of viruses with animal (except humans), plant, fungi or other eukaryotic hosts by (A) reference-based assembly or (B) *de novo* assembly in indoor air samples from a daycare centre, Belgium, January–December 2022^a^ (n = 42 samples)

Untargeted deep sequencing, combined with reference-based assembly and *de novo* assembly (Figure 4), allowed us to detect insect and animal-associated viruses in 27 of 42 samples (Figure 5). Avian species-infecting viruses, such as avian rotaviruses (detected in April, June, and October), and members of the *Gammapolyomavirus secanaria* (October) were detected. Additionally, honeybee-related viruses, such as Sacbrood virus and Moku virus (Figure 5), were identified in July and September, along with novel and divergent insect infecting densoviruses throughout the year (in 12 of 42 samples). As expected, divergent (and novel) densoviruses were only identified by *de novo* assembly, while viruses with low read counts but high similarity to known references, such as avian rotaviruses, were only identified by reference-based assembly.

f: family g: genus, s: species, sf: subfamily.

^a^ The daycare centre was closed between mid-July and September, so no samples were collected during this period. Dates with no sampling including all the month of August 2022 are omitted from the graph.

^b^ D: December.

On the X-axis, the first and last sample collection in each month is visible and the Y-axis represents viruses at taxonomical level.

**Figure 6:**
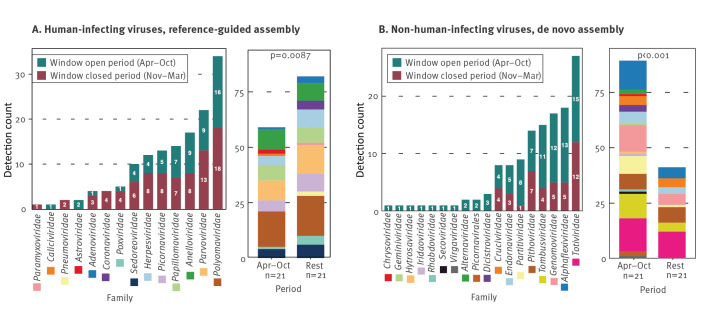
Number detections by untargeted metagenomics of different viruses in the indoor air of a daycare, when windows were open or closed, stratified according to (A) human- and (B) non-human infecting viruses, Belgium, January–December 2022

The daycare centre typically kept its windows open from April to October (metadata can be found as Supplementary information 2 S4). Therefore, we categorised detections into two periods: April–October and the rest of the year. This categorisation revealed a significantly higher prevalence of human-infecting viruses during the closed-window periods, while non-human-infecting viruses were more prevalent during the open-window period (p < 0.01, Figure 6). 

df: degrees of freedom.

A. Number of detections of human-infecting viruses for each family (panel A: graph on the left) during period with open or closed windows. Aggregated detection counts of human-infecting viruses for the periods between April–October and rest of the year (panel A: graph on the right), X^2^ = 6.86, df = 1, p = 0.0087*.* B. Number of detections of non-human-infecting viruses for each family (panel B: graph on the left). Aggregated detection counts of non-human-infecting viruses for the periods between April–October and rest of the year (panel B: graph on the right), X^2^ = 33.985, df = 1, p < *0.001.*

Additionally, we detected totiviruses, matching with *Malasseziacea* (fungi) reads, suggesting that these viruses were *Malasseziacea*-associated, as further explained in Supplementary information 2 S5. We conducted correlation coefficient analyses on environmental surveillance data to investigate whether any environmental parameters could serve as proxy for the species identified per sample, which did not reveal any significant results (p > 0.05, analyses and further explanation can be found in Supplementary information 2 S6).

## Discussion

In this study, we employed longitudinal indoor air sampling and untargeted viral metagenomics over a 1-year period at a daycare centre. We used the NetoVIR protocol to enrich in virus-like particle (VLP), combined with short-read deep sequencing, to enhance our ability to detect low-abundance viral species with greater sensitivity. Throughout the 12-month period, we successfully uncovered a diverse array of human-associated viruses, including respiratory, enteric, and skin viruses, alongside non-human-associated viruses, which were linked to animals, plants, and fungi. Additionally, we conducted an in-depth bioinformatics analysis, combining *de novo* assembly and reference-guided assembly techniques. These methods led us to uncover not only known viruses with very low abundance, but also novel and divergent viruses, making it potentially useful to identify ‘pathogen X’ — an epidemic or pandemic viral pathogen that is not yet characterised [49].

Rosario et al. previously observed Merkel cell polyomavirus, MW polyomavirus and papillomaviruses in air filter samples collected in dormitory rooms [50]. Similarly, polyomaviruses were the most abundant human viruses revealed through our work, including WU polyomavirus, a respiratory virus reported in immunocompromised people and in children with minimal or no respiratory symptoms [51,52]. We also detected MW polyomavirus as well as Merkel cell polyomavirus, both also prior documented among asymptomatic individuals [53,54]. Interestingly, alongside the human viruses we found, the most abundant fungal viruses were related to commensal human skin fungi from the family *Malasseziaceae*, providing evidence for the high abundance of human skin-associated microorganisms in the environment. Therefore, our findings suggest a potential of indoor air sampling for surveillance of skin-associated viruses.

Several recent studies have investigated the potential of indoor air surveillance to monitor specific viral pathogen dynamics and epidemiology. These were conducted in settings such as hospitals, offices, and residential environments [10,11,13,25,55-58]. All of them relied on targeted tests requiring specific viral genomic knowledge, while we employed a non-targeted approach. The longitudinal design of our investigation, in combination with available epidemiological data from the University Hospitals Leuven and NRCs allowed us to use rhinovirus and rotavirus as examples to showcase the power of air sampling in epidemiological surveillance, especially for asymptomatic circulation in a setting with no clinically ill individuals. In addition, our NGS-based untargeted methodology allowed us to subtype rhinoviruses, showing the co-circulation of various rhinoviruses throughout the year.

In a non-longitudinal study, Minor et al. examined indoor air from multiple locations with metagenomics, detecting influenza C in a preschool and providing genomic data for this frequently untested virus [25]. We expanded their approach by seeking multiple complete or partial genomes of viruses, which may be associated with respiratory (e.g. WU polyomavirus) or skin diseases but are not currently included in the routine qPCR panels for respiratory diseases in hospitals. Additionally, we were able to identify and subtype several viruses (e.g. bocaviruses, RSV) and distinguish the co-circulation of closely related virus variants (WU polyomavirus) over time. This underscores the possible simultaneous use of shotgun metagenomics to determine the presence, frequency and changing genomic/epidemiological characteristics of multiple viruses.

Beyond surveillance of human viruses, indoor air and environmental surveillance with metagenomics could also be of interest for animal farms and greenhouses. Kwok et al. analysed dust samples and pooled chicken faeces from farms using shotgun metagenomics, identifying multiple viruses in dust that matched those found in the faeces from the same location [59]. In our study, we unexpectedly detected avian viruses (rotaviruses and a divergent polyomavirus), a cat papillomavirus, and several insect-infecting viruses, including a novel densovirus, in an animal-free environment. We suggest that the animal and plant viruses mentioned are highly resilient in the environment and may be transported indoors by insects, air currents, or even on human skin or garments that can subsequently shed them. Additionally, the presence of multiple plant viruses demonstrates that indoor air can serve as an effective medium for detecting pathogens that typically require labour-intensive sampling from various crops. Therefore, we propose that indoor air surveillance in strategic animal and plant production locations can be powerful for revealing epidemiological trends and potential outbreaks of viruses.

It was previously reported that indoor air shotgun metagenomics with other methods resulted in 1% to 3.2% of reads that could be assigned to viruses [60], underscoring the low viral biomass of indoor air samples. However, using the NetoVIR protocol, 10.9% of reads mapped to viral contigs, primarily phages, aligning with earlier findings [61]. Interestingly, shotgun metagenomics identified a broader diversity of enteric viruses, such as rotavirus and sapovirus, compared with the enteric qPCR panel, whereas the respiratory qPCR panel detected rhinovirus, RSV, adenovirus, and parainfluenza more consistently than metagenomics. These differences likely stem from the distinct sensitivities of the qPCR panels, which are tailored for detecting specific viral loads in defined sample types. The viral load in indoor air is likely at or near detection thresholds, with commonly circulating human viruses comprising less than 0.005% of total reads, as reported by Prussin et al. [61]. In our study, we confirm this low viral load of human-associated viruses in indoor air apart from skin- and respiratory-associated polyomaviruses.

Given the longitudinal nature of our sample collection, it is possible to discuss seasonality of viruses. Prussin et al. employed passive indoor air sampling and shotgun metagenomics throughout the year in a daycare centre in Virginia, US. They found human-associated viruses to be much more diverse and dominant in the winter, while the summertime virome contained a higher proportion and diversity of plant-associated viruses [61]. Similarly, we also observed a higher prevalence of animal- or plant-associated viruses, particularly during the warmer months from April to October when windows were open and respiratory and enteric viruses mostly during the wintertime when windows were closed. Therefore, we suggest that active indoor air sampling can reflect dynamics of both the inside and outside environment. On the other hand, human parainfluenza virus 3 was detected in winter by indoor air sampling at the daycare centre, which is different than its defined peak in the literature, late spring and early summer.

Overall, untargeted viral metagenomics offers a strong and complementary tool for pathogen surveillance via indoor air, particularly in cases of suspected asymptomatic shedding. In practice, we would propose a surveillance system where samples are collected at regular intervals from a small number of strategically selected sentinel locations — ideally in ventilation shafts to capture air from multiple indoor spaces in each sample while further minimising the invasiveness of such sampling approach for the community [14]. This approach can leverage established specialised laboratory infrastructure and standardised workflows; with declining sequencing costs due to pooled/batched sample processing. This could result in early signals of beginning epidemic trends of seasonal, emerging and re-emerging pathogens, while at the same time providing genetic information for further characterisation. Timely information collected on viral (co-) circulation at the community level can be used to prepare the healthcare system for viral circulation. Moreover, metagenomics enables detailed genomic analyses and identification variants, which may eventually serve as a proxy for estimating the number of infected hosts or introductory events at a specific location. Future research should validate this approach at sentinel locations for skin, enteric, and respiratory viruses in targeted settings, ideally incorporating clinical data of individuals.

Our study has several limitations. Primarily, it was conducted at a single location, without access to information regarding the health status or medical history of individuals in the vicinity. Nonetheless, it was assumed that individuals were unlikely to exhibit severe respiratory or gastroenteritis-related symptoms, as it was recommended that families keep children at home if they displayed such symptoms. Secondly, we could not acquire complete genomes for numerous viruses that were present, which may stem from some methodological constraints of sequencing and low viral loads in the air. Additionally, our results are not confirmed by culture-based techniques to determine if the detected viral genomes are from infectious viruses or not. This would be important information, with respect to the potential of airborne transmission of skin-associated and enteric viruses, since dose-risk relationships of many viruses are not well understood. On the other hand, we employed a longitudinal approach over a 12-month period, allowing to monitor seasonal dynamics and use advanced viral enrichment methods with comprehensive analyses, which enhanced our ability to detect even low-abundance viral species with greater sensitivity.

## Conclusion

Our study highlights that indoor air sampling employed with viral metagenomics is a robust method for surveillance of both human-associated and non-human-associated viruses. We suggest that this method, implemented in sentinel locations and targeted populations, can be complementary to traditional patient-based surveillance methods.

## Data Availability

Raw data, excluding human reads, is available at the Sequence Read Archive (SRA) with Bioproject number PRJNA1158979. Accession numbers for respective sequences given in the manuscript originate from GenBank and can be found at https://www.ncbi.nlm.nih.gov/genbank/. Codes used to conduct the analyses and produce figures are available on https://github.com/Matthijnssenslab/Indoorair_daycare.

## Supplementary Data

226000 SupplementaryInformation1

## Supplementary Data

2228000 SupplementaryInformation2
