# Supplementary material for: Shotgun metagenomics on indoor air for surveillance of respiratory, enteric, and skin viruses in a Belgian daycare setting, January to December 2022
Source: Euro Surveill. 2025 Sep 25;30(38):2400711. doi: 10.2807/1560-7917.ES.2025.30.38.2400711 (PMC12475893; doi:10.2807/1560-7917.ES.2025.30.38.2400711)
Supplement: SupplementaryInformation1 [file 24-00711_SupplementaryInformation_1.pdf]

This supplementary material is hosted by Eurosurveillance as supporting information alongside the article "Shotgun metagenomics on indoor air for surveillance of respiratory, enteric, and skin viruses in a Belgian daycare setting, January to December 2022", on behalf of the authors, who remain responsible for the accuracy and appropriateness of the content. The same standards for ethics, copyright, attributions and permissions as for the article apply. Supplements are not edited by *Eurosurveillance* and the journal is not responsible for the maintenance of any links or email addresses provided therein.

Supplementary information 1. Obtained consensus sequences and their information

This supplementary material is hosted by Eurosurveillance as supporting information alongside the article "Shotgun metagenomics on indoor air for surveillance of respiratory, enteric, and skin viruses in a Belgian daycare setting, January to December 2022", on behalf of the authors, who remain responsible for the accuracy and appropriateness of the content. The same standards for ethics, copyright, attributions and permissions as for the article apply. Supplements are not edited by *Eurosurveillance* and the journal is not responsible for the maintenance of any links or email addresses provided therein.

Sample collection date	accession	Covered bases	Reference length	Completeness (%)	# reads aligned	Total # reads	family	species	subspecies	Respiratory panel CT-Value	Enteric panel CT-value	# people	Sample or Control
**10/01/2022**	KY316160.1	385	34169	1.13	18	13662430	Adenoviridae	Human mastadenovirus F	Human adenovirus 41	31.1	37,1	15	Sample
**10/01/2022**	EF582385.1	245	7099	3.45	27	13662430	Picornaviridae	Rhinovirus C	rhinovirus C4	32.2	NA	15	Sample
**10/01/2022**	AB041007.1	915	3852	23.75	69	13662430	Anelloviridae	Torque teno virus 1	unclassified Torque teno virus 1 subspecies/strain	NA	NA	15	Sample
**10/01/2022**	DQ915164.2	534	31076	1.72	80	13662430	Coronaviridae	Betacoronavirus 1	HCoV- OC43	33.4	NA	15	Sample
**10/01/2022**	KY490071.1	1270	235397	0.54	90	13662430	Herpesviridae	Human betaherpesvirus 5	Human betaherpesvirus 5 subspecies/strain	31.1	NA	15	Sample
**10/01/2022**	NC_007455.1	1150	5299	21.7	142	13662430	Parvoviridae	Primate bocaparvovirus 1	unclassified Primate bocaparvovirus 1 subspecies/strain	29.3	NA	15	Sample
**10/01/2022**	JX463183.1	3136	4776	65.66	619	13662430	Polyomaviridae	Deltapolyomavirus undecihominis	STL polyomavirus	NA	NA	15	Sample
**10/01/2022**	AF043303.1	4180	4679	89.34	1583	13662430	Parvoviridae	Adeno-associated dependoparvovirus A	adeno-associated virus 2	NA	NA	15	Sample
**10/01/2022**	JX262162.1	4939	4939	100	2643	13662430	Polyomaviridae	Deltapolyomavirus decihominis	unclassified Deltapolyomavirus decihominis subspecies/strain	NA	NA	15	Sample
**19/01/2022**	JQ898291.1	227	4927	4.61	16	15419422	Polyomaviridae	Deltapolyomavirus decihominis	MW polyomavirus	NA	NA	6	Sample
**19/01/2022**	JX262162.1	2269	4939	45.94	461	15419422	Polyomaviridae	Deltapolyomavirus decihominis	unclassified Deltapolyomavirus decihominis subspecies/strain	NA	NA	6	Sample
**24/01/2022**	AB054647.1	203	3790	5.36	2	6572238	Anelloviridae	Torque teno virus 8	unclassified Torque teno virus 8 subspecies/strain	NA	NA	10	Sample
**24/01/2022**	JQ898291.1	324	4927	6.58	3	6572238	Polyomaviridae	Deltapolyomavirus decihominis	MW polyomavirus	NA	NA	10	Sample
**24/01/2022**	AF043303.1	773	4679	16.52	44	6572238	Parvoviridae	Adeno-associated dependoparvovirus A	adeno-associated virus 2	NA	NA	10	Sample
**24/01/2022**	JX262162.1	3972	4939	80.42	376	6572238	Polyomaviridae	Deltapolyomavirus decihominis	unclassified Deltapolyomavirus decihominis subspecies/strain	NA	NA	10	Sample
**26/01/2022**	KY490071.1	231	235397	0.1	9	19014920	Herpesviridae	Human betaherpesvirus 5	unclassified Human betaherpesvirus 5 subspecies/strain	29.6	NA	10	Sample
**26/01/2022**	KY040275.1	1385	188253	0.74	17	19014920	Poxviridae	Molluscum contagiosum virus	Molluscum contagiosum virus subtype 1	NA	NA	10	Sample

This supplementary material is hosted by Eurosurveillance as supporting information alongside the article "Shotgun metagenomics on indoor air for surveillance of respiratory, enteric, and skin viruses in a Belgian daycare setting, January to December 2022", on behalf of the authors, who remain responsible for the accuracy and appropriateness of the content. The same standards for ethics, copyright, attributions and permissions as for the article apply. Supplements are not edited by *Eurosurveillance* and the journal is not responsible for the maintenance of any links or email addresses provided therein.

**26/01/2022**	NC_001781.1	400	15225	2.63	46	19014920	Pneumoviridae	Human orthopneumovirus	unclassified Human orthopneumovirus subspecies/strain	35.7	NA	10	Sample
**26/01/2022**	JX262162.1	1575	4939	31.89	260	19014920	Polyomaviridae	Deltapolyomavirus decihominis	unclassified Deltapolyomavirus decihominis subspecies/strain	NA	NA	10	Sample
**26/01/2022**	NC_007455.1	2161	5299	40.78	292	19014920	Parvoviridae	Primate bocaparvovirus 1	unclassified Primate bocaparvovirus 1 subspecies/strain	32.7	NA	10	Sample
**26/01/2022**	AF043303.1	3073	4679	65.68	1229	19014920	Parvoviridae	Adeno-associated dependoparvovirus A	adeno-associated virus 2	NA	NA	10	Sample
**31/01/2022**	KC782520.2	163	2687	6.07	2	16984456	Sedoreoviridae	Rotavirus A		NA	*Not detected*	15	Sample
**31/01/2022**	KC782515.2	119	1059	11.24	4	16984456	Sedoreoviridae	Rotavirus A		NA	*Not detected*	15	Sample
**31/01/2022**	EF059923.1	372	2359	15.77	6	16984456	Sedoreoviridae	Rotavirus A		NA	*Not detected*	15	Sample
**31/01/2022**	KC442988.1	192	2333	8.23	7	16984456	Sedoreoviridae	Rotavirus A		NA	*Not detected*	15	Sample
**31/01/2022**	MG181725.1	286	2359	12.12	9	16984456	Sedoreoviridae	Rotavirus A		NA	*Not detected*	15	Sample
**31/01/2022**	JX134046.1	356	2915	12.21	11	16984456	Anelloviridae	TTV-like mini virus	unclassified TTV-like mini virus subspecies/strain	NA	NA	15	Sample
**31/01/2022**	AF261761.1	365	3736	9.77	36	16984456	Anelloviridae	Torque teno virus 7	unclassified Torque teno virus 7 subspecies/strain	NA	NA	15	Sample
**31/01/2022**	NC_007455.1	238	5299	4.49	38	16984456	Parvoviridae	Primate bocaparvovirus 1	unclassified Primate bocaparvovirus 1 subspecies/strain	30.3	NA	15	Sample
**31/01/2022**	JX463183.1	564	4776	11.81	41	16984456	Polyomaviridae	Deltapolyomavirus undecihominis	STL polyomavirus	NA	NA	15	Sample
**31/01/2022**	EU326526.1	636	15462	4.11	70	16984456	Paramyxoviridae	Human respirovirus 3	unclassified Human respirovirus 3 subspecies/strain	37.2	NA	15	Sample
**31/01/2022**	AB060594.1	740	3234	22.88	130	16984456	Anelloviridae	Torque teno virus 20	unclassified Torque teno virus 20 subspecies/strain	NA	NA	15	Sample
**31/01/2022**	KY316160.1	2602	34169	7.62	184	16984456	Adenoviridae	Human mastadenovirus F	Human adenovirus 41	29.1	*Not detected*	15	Sample
**31/01/2022**	GQ845442.1	3512	7304	48.08	440	16984456	Papillomaviridae	Betapapillomavirus 2	Human papillomavirus 120	NA	NA	15	Sample
**31/01/2022**	JX262162.1	4014	4939	81.27	850	16984456	Polyomaviridae	Deltapolyomavirus decihominis	unclassified Deltapolyomavirus decihominis subspecies/strain	NA	NA	15	Sample
**31/01/2022**	AF043303.1	4436	4679	94.81	4396	16984456	Parvoviridae	Adeno-associated dependoparvovirus A	adeno-associated virus 2	NA	NA	15	Sample
**07/02/2022**	KJ753392.1	138	2530	5.45	2	9911346	Sedoreoviridae	Rotavirus A		NA	*Not detected*	15	Sample
**07/02/2022**	KC782514.2	265	1566	16.92	4	9911346	Sedoreoviridae	Rotavirus A		NA	*Not detected*	15	Sample

This supplementary material is hosted by Eurosurveillance as supporting information alongside the article "Shotgun metagenomics on indoor air for surveillance of respiratory, enteric, and skin viruses in a Belgian daycare setting, January to December 2022", on behalf of the authors, who remain responsible for the accuracy and appropriateness of the content. The same standards for ethics, copyright, attributions and permissions as for the article apply. Supplements are not edited by *Eurosurveillance* and the journal is not responsible for the maintenance of any links or email addresses provided therein.

**07/02/2022**	JX262162.1	257	4939	5.2	5	9911346	Polyomaviridae	Deltapolyomavirus decihominis	unclassified Deltapolyomavirus decihominis subspecies/strain	NA	NA	15	Sample
**07/02/2022**	JX463183.1	694	4776	14.53	36	9911346	Polyomaviridae	Deltapolyomavirus undecihominis	STL polyomavirus	NA	NA	15	Sample
**07/02/2022**	AF043303.1	1127	4679	24.09	62	9911346	Parvoviridae	Adeno-associated dependoparvovirus A	adeno-associated virus 2	NA	NA	15	Sample
**07/02/2022**	NC_001781.1	1199	15225	7.88	65	9911346	Pneumoviridae	Human orthopneumovirus	unclassified Human orthopneumovirus subspecies/strain	32	NA	15	Sample
**07/02/2022**	JQ898291.1	1263	4927	25.63	67	9911346	Polyomaviridae	Deltapolyomavirus decihominis	MW polyomavirus	NA	NA	15	Sample
**07/02/2022**	NC_007455.1	2339	5299	44.14	205	9911346	Parvoviridae	Primate bocaparvovirus 1	unclassified Primate bocaparvovirus 1 subspecies/strain	29.8	NA	15	Sample
**07/02/2022**	MK212031.1	1433	2907	49.29	207	9911346	Anelloviridae	TTV-like mini virus	unclassified TTV-like mini virus subspecies/strain	NA	NA	15	Sample
**21/02/2022**	JX040418.1	118	750	15.73	4	19682352	Sedoreoviridae	Rotavirus A		NA	*Not detected*	20	Sample
**21/02/2022**	KU727766.1	563	5166	10.9	24	19682352	Parvoviridae	Parus major densovirus	unclassified Parus major densovirus subspecies/strain	NA	NA	20	Sample
**21/02/2022**	NC_012564.1	391	5242	7.46	64	19682352	Parvoviridae	Primate bocaparvovirus 1	Human bocavirus 3	29.6	NA	20	Sample
**21/02/2022**	AF043303.1	1394	4679	29.79	87	19682352	Parvoviridae	Adeno-associated dependoparvovirus A	adeno-associated virus 2	NA	NA	20	Sample
**21/02/2022**	EU796884.1	1333	7899	16.88	121	19682352	Papillomaviridae	Dyothetapapillomavirus 1	Felis domesticus papillomavirus 2	NA	NA	20	Sample
**21/02/2022**	NC_007455.1	1293	5299	24.4	132	19682352	Parvoviridae	Primate bocaparvovirus 1	unclassified Primate bocaparvovirus 1 subspecies/strain	29.6	NA	20	Sample
**21/02/2022**	KY490071.1	5617	235397	2.39	345	19682352	Herpesviridae	Human betaherpesvirus 5	unclassified Human betaherpesvirus 5 subspecies/strain	28.9	NA	20	Sample
**21/02/2022**	JX262162.1	4059	4939	82.18	685	19682352	Polyomaviridae	Deltapolyomavirus decihominis	unclassified Deltapolyomavirus decihominis subspecies/strain	NA	NA	20	Sample
**23/02/2022**	JQ898291.1	252	4927	5.11	10	13805648	Polyomaviridae	Deltapolyomavirus decihominis	MW polyomavirus	NA	NA	16	Sample
**23/02/2022**	AF043303.1	857	4679	18.32	98	13805648	Parvoviridae	Adeno-associated dependoparvovirus A	adeno-associated virus 2	NA	NA	16	Sample
**23/02/2022**	KT779555.1	1102	29887	3.69	107	13805648	Coronaviridae	Human coronavirus HKU1	unclassified Human coronavirus HKU1 subspecies/strain	27.9	NA	16	Sample
**23/02/2022**	GU233853.1	1947	7219	26.97	306	13805648	Papillomaviridae	Gammapapillomavirus 9	Human papillomavirus type 129	NA	NA	16	Sample

This supplementary material is hosted by Eurosurveillance as supporting information alongside the article "Shotgun metagenomics on indoor air for surveillance of respiratory, enteric, and skin viruses in a Belgian daycare setting, January to December 2022", on behalf of the authors, who remain responsible for the accuracy and appropriateness of the content. The same standards for ethics, copyright, attributions and permissions as for the article apply. Supplements are not edited by *Eurosurveillance* and the journal is not responsible for the maintenance of any links or email addresses provided therein.

**23/02/2022**	JX262162.1	938	4939	18.99	483	13805648	Polyomaviridae	Deltapolyomavirus decihominis	unclassified Deltapolyomavirus decihominis subspecies/strain	NA	NA	16	Sample
**04/03/2022**	JQ898291.1	391	4927	7.94	5	14696626	Polyomaviridae	Deltapolyomavirus decihominis	MW polyomavirus	NA	NA	17	Sample
**04/03/2022**	KY629935.1	317	7130	4.45	15	14696626	Picornaviridae	Rhinovirus A	rhinovirus A59	34.3	NA	17	Sample
**04/03/2022**	KT779555.1	334	29887	1.12	19	14696626	Coronaviridae	Human coronavirus HKU1	unclassified Human coronavirus HKU1 subspecies/strain	30.3	NA	17	Sample
**04/03/2022**	KY490071.1	643	235397	0.27	26	14696626	Herpesviridae	Human betaherpesvirus 5	unclassified Human betaherpesvirus 5 subspecies/strain	29.8	NA	17	Sample
**04/03/2022**	KY040275.1	1039	188253	0.55	33	14696626	Poxviridae	Molluscum contagiosum virus	Molluscum contagiosum virus subtype 1	NA	NA	17	Sample
**04/03/2022**	GU233853.1	710	7219	9.84	59	14696626	Papillomaviridae	Gammapapillomavirus 9	Human papillomavirus type 129	NA	NA	17	Sample
**04/03/2022**	AF043303.1	2569	4679	54.9	174	14696626	Parvoviridae	Adeno-associated dependoparvovirus A	adeno-associated virus 2	NA	NA	17	Sample
**04/03/2022**	JX262162.1	3188	4939	64.55	310	14696626	Polyomaviridae	Deltapolyomavirus decihominis	unclassified Deltapolyomavirus decihominis subspecies/strain	NA	NA	17	Sample
**07/03/2022**	KM254174.1	236	3661	6.45	4	12801052	Parvoviridae	Galliform chaphamaparvovirus 3	chicken chapparvovirus HK	NA	NA	23	Sample
**07/03/2022**	AB054647.1	553	3790	14.59	8	12801052	Anelloviridae	Torque teno virus 8	unclassified Torque teno virus 8 subspecies/strain	NA	NA	23	Sample
**07/03/2022**	KC782516.2	117	1066	10.98	8	12801052	Sedoreoviridae	Rotavirus A		NA	*Not detected*	23	Sample
**07/03/2022**	JX262162.1	439	4939	8.89	24	12801052	Polyomaviridae	Deltapolyomavirus decihominis	unclassified Deltapolyomavirus decihominis subspecies/strain	NA	NA	23	Sample
**07/03/2022**	NC_012564.1	414	5242	7.9	43	12801052	Parvoviridae	Primate bocaparvovirus 1	Human bocavirus 3	34.4	NA	23	Sample
**09/03/2022**	NC_012564.1	356	5242	6.79	14	16130390	Parvoviridae	Primate bocaparvovirus 1	Human bocavirus 3	33.6	NA	18	Sample
**09/03/2022**	JX463183.1	713	4776	14.93	15	16130390	Polyomaviridae	Deltapolyomavirus undecihominis	STL polyomavirus	NA	NA	18	Sample
**09/03/2022**	MG846442.1	496	4432	11.19	21	16130390	Parvoviridae	Galliform chaphamaparvovirus 2	Chicken chapparvovirus 2	NA	NA	18	Sample
**09/03/2022**	JX262162.1	1072	4939	21.7	48	16130390	Polyomaviridae	Deltapolyomavirus decihominis	unclassified Deltapolyomavirus decihominis subspecies/strain	NA	NA	18	Sample
**11/03/2022**	KY629935.1	557	7130	7.81	21	19895646	Picornaviridae	Rhinovirus A	rhinovirus A59	31	NA	23	Sample
**11/03/2022**	HM011556.1	838	5387	15.56	22	19895646	Polyomaviridae	Alphapolyomavirus quintihominis	Merkel cell polyomavirus	NA	NA	23	Sample

This supplementary material is hosted by Eurosurveillance as supporting information alongside the article "Shotgun metagenomics on indoor air for surveillance of respiratory, enteric, and skin viruses in a Belgian daycare setting, January to December 2022", on behalf of the authors, who remain responsible for the accuracy and appropriateness of the content. The same standards for ethics, copyright, attributions and permissions as for the article apply. Supplements are not edited by *Eurosurveillance* and the journal is not responsible for the maintenance of any links or email addresses provided therein.

**11/03/2022**	JX262162.1	1816	4939	36.77	99	19895646	Polyomaviridae	Deltapolyomavirus decihominis	unclassified Deltapolyomavirus decihominis subspecies/strain	NA	NA	23	Sample
**14/03/2022**	MF898328.1	234	125095	0.19	10	12667232	Herpesviridae	Human alphaherpesvirus 3	unclassified Human alphaherpesvirus 3 subspecies/strain	NA	NA	21	Sample
**14/03/2022**	KY629935.1	420	7130	5.89	40	12667232	Picornaviridae	Rhinovirus A	rhinovirus A59	33.1	NA	21	Sample
**14/03/2022**	JX262162.1	4323	4939	87.53	1033	12667232	Polyomaviridae	Deltapolyomavirus decihominis	unclassified Deltapolyomavirus decihominis subspecies/strain	NA	NA	21	Sample
**16/03/2022**	JX262162.1	461	4939	9.33	9	13284002	Polyomaviridae	Deltapolyomavirus decihominis	unclassified Deltapolyomavirus decihominis subspecies/strain	NA	NA	20	Sample
**16/03/2022**	GU233853.1	2329	7219	32.26	76	13284002	Papillomaviridae	Gammapapillomavirus 9	Human papillomavirus type 129	NA	NA	20	Sample
**21/03/2022**	KY629935.1	331	7130	4.64	12	19633702	Picornaviridae	Rhinovirus A	rhinovirus A59	34.6	NA	22	Sample
**21/03/2022**	MG846442.1	387	4432	8.73	18	19633702	Parvoviridae	Galliform chaphamaparvovirus 2	Chicken chapparvovirus 2	NA	NA	22	Sample
**21/03/2022**	MF588730.1	212	7292	2.91	24	19633702	Papillomaviridae	Gammapapillomavirus 18	unclassified Gammapapillomavirus 18 subspecies/strain	NA	NA	22	Sample
**23/03/2022**	FJ169853.1	142	3305	4.3	8	16564138	Sedoreoviridae	Rotavirus A		NA	*Not detected*	18	Sample
**23/03/2022**	KY629935.1	353	7130	4.95	18	16564138	Picornaviridae	Rhinovirus A	rhinovirus A59	34.5	NA	18	Sample
**23/03/2022**	KU569162.1	686	5154	13.31	38	16564138	Parvoviridae	Galliform aveparvovirus 1	unclassified Galliform aveparvovirus 1 subspecies/strain	NA	NA	18	Sample
**23/03/2022**	JX262162.1	1732	4939	35.07	108	16564138	Polyomaviridae	Deltapolyomavirus decihominis	unclassified Deltapolyomavirus decihominis subspecies/strain	NA	NA	18	Sample
**30/03/2022**	KC782520.2	124	2687	4.61	6	19652094	Sedoreoviridae	Rotavirus A			*Not detected*	17	Sample
**30/03/2022**	X74468.1	210	7412	2.83	13	19652094	Papillomaviridae	Betapapillomavirus 2	Human papillomavirus 15	NA	NA	17	Sample
**30/03/2022**	MF898328.1	437	125095	0.35	14	19652094	Herpesviridae	Human alphaherpesvirus 3	unclassified Human alphaherpesvirus 3 subspecies/strain	NA	NA	17	Sample
**30/03/2022**	AB017613.1	246	3818	6.44	34	19652094	Anelloviridae	Torque teno virus 16	unclassified Torque teno virus 16 subspecies/strain	NA	NA	17	Sample
**30/03/2022**	GU233853.1	548	7219	7.59	58	19652094	Papillomaviridae	Gammapapillomavirus 9	Human papillomavirus type 129	NA	NA	17	Sample
**30/03/2022**	FJ349096.1	2158	35758	6.04	71	19652094	Adenoviridae	Human mastadenovirus C		31.8	NA	17	Sample
**30/03/2022**	KY629935.1	345	7130	4.84	85	19652094	Picornaviridae	Rhinovirus A	rhinovirus A59	*Not detected*	NA	17	Sample
**30/03/2022**	X74464.1	1700	7434	22.87	93	19652094	Papillomaviridae	Betapapillomavirus 2	Human papillomavirus 9	NA	NA	17	Sample

This supplementary material is hosted by Eurosurveillance as supporting information alongside the article "Shotgun metagenomics on indoor air for surveillance of respiratory, enteric, and skin viruses in a Belgian daycare setting, January to December 2022", on behalf of the authors, who remain responsible for the accuracy and appropriateness of the content. The same standards for ethics, copyright, attributions and permissions as for the article apply. Supplements are not edited by *Eurosurveillance* and the journal is not responsible for the maintenance of any links or email addresses provided therein.

**30/03/2022**	AC_000008.1	1784	35938	4.96	108	19652094	Adenoviridae	Human mastadenovirus C		31.8	NA	17	Sample
**30/03/2022**	AF261761.1	870	3736	23.29	163	19652094	Anelloviridae	Torque teno virus 7	unclassified Torque teno virus 7 subspecies/strain	NA	NA	17	Sample
**30/03/2022**	AC_000017.1	2926	36001	8.13	175	19652094	Adenoviridae	Human mastadenovirus C		31.8	NA	17	Sample
**30/03/2022**	JX262162.1	3172	4939	64.22	598	19652094	Polyomaviridae	Deltapolyomavirus decihominis	unclassified Deltapolyomavirus decihominis subspecies/strain	NA	NA	17	Sample
**30/03/2022**	KY490071.1	12244	235397	5.2	998	19652094	Herpesviridae	Human betaherpesvirus 5	unclassified Human betaherpesvirus 5 subspecies/strain	28.8	NA	17	Sample
**04/04/2022**	JX262162.1	531	4939	10.75	17	15622914	Polyomaviridae	Deltapolyomavirus decihominis	unclassified Deltapolyomavirus decihominis subspecies/strain	NA	NA	15	Sample
**08/04/2022**	HM011556.1	260	5387	4.83	3	12603994	Polyomaviridae	Alphapolyomavirus quintihominis	Merkel cell polyomavirus	NA	NA	16	Sample
**08/04/2022**	MG846442.1	237	4432	5.35	7	12603994	Parvoviridae	Galliform chaphamaparvovirus 2	Chicken chapparvovirus 2	NA	NA	16	Sample
**08/04/2022**	AB041962.1	263	2908	9.04	15	12603994	Anelloviridae	Torque teno mini virus 5	unclassified Torque teno mini virus 5 subspecies/strain	NA	NA	16	Sample
**08/04/2022**	AB017613.1	273	3818	7.15	20	12603994	Anelloviridae	Torque teno virus 16	unclassified Torque teno virus 16 subspecies/strain	NA	NA	16	Sample
**08/04/2022**	AF043303.1	1425	4679	30.46	133	12603994	Parvoviridae	Adeno-associated dependoparvovirus A	adeno-associated virus 2	NA	NA	16	Sample
**08/04/2022**	JX262162.1	2563	4939	51.89	334	12603994	Polyomaviridae	Deltapolyomavirus decihominis	unclassified Deltapolyomavirus decihominis subspecies/strain	NA	NA	16	Sample
**08/04/2022**	NC_007455.1	4768	5299	89.98	1070	12603994	Parvoviridae	Primate bocaparvovirus 1	unclassified Primate bocaparvovirus 1 subspecies/strain	26.9	NA	16	Sample
**25/04/2022**	KU356638.1	190	751	25.3	5	9122068	Sedoreoviridae	Rotavirus A		NA	*Not detected*	22	Sample
**25/04/2022**	KY490071.1	241	235397	0.1	8	9122068	Herpesviridae	Human betaherpesvirus 5	unclassified Human betaherpesvirus 5 subspecies/strain	30	NA	22	Sample
**25/04/2022**	KU727766.1	232	5166	4.49	10	9122068	Parvoviridae	Parus major densovirus	unclassified Parus major densovirus subspecies/strain	NA	NA	22	Sample
**25/04/2022**	FJ445146.1	420	7141	5.88	30	9122068	Picornaviridae	Rhinovirus A	rhinovirus A63	32.1	NA	22	Sample
**25/04/2022**	NC_007455.1	844	5299	15.93	97	9122068	Parvoviridae	Primate bocaparvovirus 1	unclassified Primate bocaparvovirus 1 subspecies/strain	28.5	NA	22	Sample
**25/04/2022**	JX262162.1	3373	4939	68.29	334	9122068	Polyomaviridae	Deltapolyomavirus decihominis	unclassified Deltapolyomavirus decihominis subspecies/strain	NA	NA	22	Sample
**27/04/2022**	KJ753392.1	190	2530	7.51	3	9993384	Sedoreoviridae	Rotavirus A		NA	*Not detected*	18	Sample
**27/04/2022**	KC443588.1	155	2650	5.85	3	9993384	Sedoreoviridae	Rotavirus A		NA	*Not detected*	18	Sample

This supplementary material is hosted by Eurosurveillance as supporting information alongside the article "Shotgun metagenomics on indoor air for surveillance of respiratory, enteric, and skin viruses in a Belgian daycare setting, January to December 2022", on behalf of the authors, who remain responsible for the accuracy and appropriateness of the content. The same standards for ethics, copyright, attributions and permissions as for the article apply. Supplements are not edited by *Eurosurveillance* and the journal is not responsible for the maintenance of any links or email addresses provided therein.

**27/04/2022**	NC_021583.1	271	1295	20.93	4	9993384	Sedoreoviridae	Rotavirus G		NA	NA	18	Sample
**27/04/2022**	KP882477.1	314	2508	12.52	4	9993384	Sedoreoviridae	Rotavirus A		NA	*Not detected*	18	Sample
**27/04/2022**	KJ752082.1	132	834	15.83	8	9993384	Sedoreoviridae	Rotavirus G		NA	NA	18	Sample
**27/04/2022**	KC178774.1	211	2687	7.85	10	9993384	Sedoreoviridae	Rotavirus A		NA	*Not detected*	18	Sample
**27/04/2022**	NC_007455.1	294	5299	5.55	10	9993384	Parvoviridae	Primate bocaparvovirus 1	unclassified Primate bocaparvovirus 1 subspecies/strain	29.9	NA	18	Sample
**27/04/2022**	GU214704.1	241	5257	4.58	20	9993384	Parvoviridae	Galliform aveparvovirus 1	Chicken parvovirus ABU-P1	NA	NA	18	Sample
**27/04/2022**	KC782520.2	485	2687	18.05	21	9993384	Sedoreoviridae	Rotavirus A		NA	*Not detected*	18	Sample
**27/04/2022**	KY055429.1	925	2585	35.78	29	9993384	Sedoreoviridae	Rotavirus A		NA	*Not detected*	18	Sample
**27/04/2022**	MG846442.1	347	4432	7.83	33	9993384	Parvoviridae	Galliform chaphamaparvovirus 2	Chicken chapparvovirus 2	NA	NA	18	Sample
**27/04/2022**	KC782514.2	256	1566	16.35	40	9993384	Sedoreoviridae	Rotavirus A		NA	*Not detected*	18	Sample
**27/04/2022**	JX262162.1	2467	4939	49.95	213	9993384	Polyomaviridae	Deltapolyomavirus decihominis	unclassified Deltapolyomavirus decihominis subspecies/strain	NA	NA	18	Sample
**11/05/2022**	KY490071.1	201	235397	0.09	16	18147382	Herpesviridae	Human betaherpesvirus 5	unclassified Human betaherpesvirus 5 subspecies/strain	29.8	NA	20	Sample
**11/05/2022**	FJ755404.1	728	6771	10.75	34	18147382	Astroviridae	Mamastrovirus 1	Human astrovirus 1 Beijing/291/2007/CHN	NA	37.9	20	Sample
**11/05/2022**	JX262162.1	678	4939	13.73	38	18147382	Polyomaviridae	Deltapolyomavirus decihominis	unclassified Deltapolyomavirus decihominis subspecies/strain	NA	NA	20	Sample
**11/05/2022**	GU233853.1	1166	7219	16.15	173	18147382	Papillomaviridae	Gammapapillomavirus 9	Human papillomavirus type 129	NA	NA	20	Sample
**11/05/2022**	MF684776.1	1447	6803	21.27	247	18147382	Astroviridae	Mamastrovirus 1	Human astrovirus 5	NA	37.9	20	Sample
**11/05/2022**	AY720891.1	2261	6723	33.63	493	18147382	Astroviridae	Mamastrovirus 1	Human astrovirus 4	NA	37.9	20	Sample
**18/05/2022**	EF554148.1	143	3302	4.33	30	15571202	Sedoreoviridae	Rotavirus A		NA	*Not detected*	16	Sample
**30/05/2022**	EU796884.1	296	7899	3.75	45	38552982	Papillomaviridae	Dyothetapapillomavirus 1	Felis domesticus papillomavirus 2	NA	NA	18	Sample
**30/05/2022**	MF684776.1	472	6803	6.94	131	38552982	Astroviridae	Mamastrovirus 1	Human astrovirus 5	NA	*Not detected*	18	Sample
**30/05/2022**	AY720891.1	637	6723	9.47	138	38552982	Astroviridae	Mamastrovirus 1	Human astrovirus 4	NA	*Not detected*	18	Sample
**30/05/2022**	MG881840.1	599	6992	8.57	144	38552982	Picornaviridae	Rhinovirus C	rhinovirus C15	32.3	NA	18	Sample
**30/05/2022**	JX262162.1	4731	4939	95.79	####	38552982	Polyomaviridae	Deltapolyomavirus decihominis	unclassified Deltapolyomavirus decihominis subspecies/strain	NA	NA	18	Sample

This supplementary material is hosted by Eurosurveillance as supporting information alongside the article "Shotgun metagenomics on indoor air for surveillance of respiratory, enteric, and skin viruses in a Belgian daycare setting, January to December 2022", on behalf of the authors, who remain responsible for the accuracy and appropriateness of the content. The same standards for ethics, copyright, attributions and permissions as for the article apply. Supplements are not edited by *Eurosurveillance* and the journal is not responsible for the maintenance of any links or email addresses provided therein.

**30/05/2022**	EF444549.1	5229	5229	100	####	38552982	Polyomaviridae	Betapolyomavirus quartihominis	WU Polyomavirus	NA	NA	18	Sample
**08/06/2022**	MK212031.1	344	2907	11.83	5	12284646	Anelloviridae	TTV-like mini virus	unclassified TTV-like mini virus subspecies/strain	NA	NA	18	Sample
**08/06/2022**	KY490071.1	229	235397	0.1	9	12284646	Herpesviridae	Human betaherpesvirus 5	unclassified Human betaherpesvirus 5 subspecies/strain	28.2	NA	18	Sample
**08/06/2022**	JX262162.1	266	4939	5.39	16	12284646	Polyomaviridae	Deltapolyomavirus decihominis	unclassified Deltapolyomavirus decihominis subspecies/strain	NA	NA	18	Sample
**08/06/2022**	KU727766.1	417	5166	8.07	40	12284646	Parvoviridae	Parus major densovirus	unclassified Parus major densovirus subspecies/strain	NA	NA	18	Sample
**08/06/2022**	EF444549.1	1060	5229	20.27	84	12284646	Polyomaviridae	Betapolyomavirus quartihominis	WU Polyomavirus	NA	NA	18	Sample
**15/06/2022**	MG846442.1	244	4432	5.51	3	17639852	Parvoviridae	Galliform chaphamaparvovirus 2	Chicken chapparvovirus 2	NA	NA	14	Sample
**15/06/2022**	AX174942.1	205	3847	5.33	9	17639852	Anelloviridae	Torque teno virus 22	unclassified Torque teno virus 22 subspecies/strain	NA	NA	14	Sample
**15/06/2022**	KU727766.1	354	5166	6.85	13	17639852	Parvoviridae	Parus major densovirus	unclassified Parus major densovirus subspecies/strain	NA	NA	14	Sample
**15/06/2022**	U31779.1	281	7779	3.61	14	17639852	Papillomaviridae	Betapapillomavirus 1	human papillomavirus 21	NA	NA	14	Sample
**15/06/2022**	KY490071.1	312	235397	0.13	16	17639852	Herpesviridae	Human betaherpesvirus 5	unclassified Human betaherpesvirus 5 subspecies/strain	29.9	NA	14	Sample
**15/06/2022**	JX262162.1	324	4939	6.56	18	17639852	Polyomaviridae	Deltapolyomavirus decihominis	unclassified Deltapolyomavirus decihominis subspecies/strain	NA	NA	14	Sample
**15/06/2022**	EF444549.1	5229	5229	100	####	17639852	Polyomaviridae	Betapolyomavirus quartihominis	WU Polyomavirus	NA	NA	14	Sample
**22/06/2022**	GU733444.1	131	2801	4.68	2	19761762	Sedoreoviridae	Rotavirus D		NA	NA	19	Sample
**22/06/2022**	KU569162.1	441	5154	8.56	11	19761762	Parvoviridae	Galliform aveparvovirus 1	unclassified Galliform aveparvovirus 1 subspecies/strain	NA	NA	19	Sample
**22/06/2022**	KU727766.1	348	5166	6.74	35	19761762	Parvoviridae	Parus major densovirus	unclassified Parus major densovirus subspecies/strain	NA	NA	19	Sample
**22/06/2022**	AB017613.1	670	3818	17.55	38	19761762	Anelloviridae	Torque teno virus 16	unclassified Torque teno virus 16 subspecies/strain	NA	NA	19	Sample
**22/06/2022**	JX262162.1	848	4939	17.17	41	19761762	Polyomaviridae	Deltapolyomavirus decihominis	unclassified Deltapolyomavirus decihominis subspecies/strain	NA	NA	19	Sample
**22/06/2022**	EF444549.1	5229	5229	100	2653	19761762	Polyomaviridae	Betapolyomavirus quartihominis	WU Polyomavirus	NA	NA	19	Sample
**29/06/2022**	KU727766.1	281	5166	5.44	4	12613618	Parvoviridae	Parus major densovirus	unclassified Parus major densovirus subspecies/strain	NA	NA	23	Sample

This supplementary material is hosted by Eurosurveillance as supporting information alongside the article "Shotgun metagenomics on indoor air for surveillance of respiratory, enteric, and skin viruses in a Belgian daycare setting, January to December 2022", on behalf of the authors, who remain responsible for the accuracy and appropriateness of the content. The same standards for ethics, copyright, attributions and permissions as for the article apply. Supplements are not edited by *Eurosurveillance* and the journal is not responsible for the maintenance of any links or email addresses provided therein.

**29/06/2022**	MG846442.1	394	4432	8.89	9	12613618	Parvoviridae	Galliform chaphamaparvovirus 2	Chicken chapparvovirus 2	NA	NA	23	Sample
**29/06/2022**	EF444549.1	1186	5229	22.68	49	12613618	Polyomaviridae	Betapolyomavirus quartihominis	WU Polyomavirus	NA	NA	23	Sample
**29/06/2022**	NC_007455.1	740	5299	13.96	56	12613618	Parvoviridae	Primate bocaparvovirus 1	unclassified Primate bocaparvovirus 1 subspecies/strain	32.7	NA	23	Sample
**29/06/2022**	NC_012564.1	2492	5242	47.54	227	12613618	Parvoviridae	Primate bocaparvovirus 1	Human bocavirus 3	32.7	NA	23	Sample
**06/07/2022**	NC_012042.1	240	5196	4.62	22	23622894	Parvoviridae	Primate bocaparvovirus 2	Human bocavirus 2c PK	30.6	NA	18	Sample
**06/07/2022**	U37537.1	236	7868	3	75	23622894	Papillomaviridae	Alphapapillomavirus 4	Human papillomavirus type 57b	NA	NA	18	Sample
**06/07/2022**	AB017613.1	347	3818	9.09	122	23622894	Anelloviridae	Torque teno virus 16	unclassified Torque teno virus 16 subspecies/strain	NA	NA	18	Sample
**06/07/2022**	JX262162.1	1278	4939	25.88	266	23622894	Polyomaviridae	Deltapolyomavirus decihominis	unclassified Deltapolyomavirus decihominis subspecies/strain	NA	NA	18	Sample
**06/07/2022**	NC_007455.1	2040	5299	38.5	739	23622894	Parvoviridae	Primate bocaparvovirus 1	unclassified Primate bocaparvovirus 1 subspecies/strain	30.6	NA	18	Sample
**06/07/2022**	NC_012564.1	3995	5242	76.21	3355	23622894	Parvoviridae	Primate bocaparvovirus 1	Human bocavirus 3	30.6	NA	18	Sample
**06/07/2022**	FN677756.1	6648	7386	90.01	6002	23622894	Papillomaviridae	Betapapillomavirus 2	Human papillomavirus 151	NA	NA	18	Sample
**06/07/2022**	EF444549.1	5229	5229	100	####	23622894	Polyomaviridae	Betapolyomavirus quartihominis	WU Polyomavirus	NA	NA	18	Sample
**13/07/2022**	KU727766.1	310	5166	6	12	23210736	Parvoviridae	Parus major densovirus	unclassified Parus major densovirus subspecies/strain	NA	NA	18	Sample
**13/07/2022**	AF536531.1	360	9812	3.67	28	23210736	Dicistroviridae	Aphid lethal paralysis virus	unclassified Aphid lethal paralysis virus subspecies/strain	NA	NA	18	Sample
**13/07/2022**	AF092924.1	606	8832	6.86	110	23210736	Iflaviridae	Sacbrood virus	unclassified Sacbrood virus subspecies/strain	NA	NA	18	Sample
**13/07/2022**	JX262162.1	1205	4939	24.4	195	23210736	Polyomaviridae	Deltapolyomavirus decihominis	unclassified Deltapolyomavirus decihominis subspecies/strain	NA	NA	18	Sample
**13/07/2022**	NC_007455.1	1780	5299	33.59	274	23210736	Parvoviridae	Primate bocaparvovirus 1	unclassified Primate bocaparvovirus 1 subspecies/strain	30.3	NA	18	Sample
**13/07/2022**	U31779.1	2190	7779	28.15	803	23210736	Papillomaviridae	Betapapillomavirus 1	human papillomavirus 21	NA	NA	18	Sample
**13/07/2022**	EF444549.1	3756	5229	71.83	892	23210736	Polyomaviridae	Betapolyomavirus quartihominis	WU Polyomavirus	NA	NA	18	Sample
**07/09/2022**	JQ898291.1	394	4927	8	6	27712580	Polyomaviridae	Deltapolyomavirus decihominis	MW polyomavirus	NA	NA	19	Sample

This supplementary material is hosted by Eurosurveillance as supporting information alongside the article "Shotgun metagenomics on indoor air for surveillance of respiratory, enteric, and skin viruses in a Belgian daycare setting, January to December 2022", on behalf of the authors, who remain responsible for the accuracy and appropriateness of the content. The same standards for ethics, copyright, attributions and permissions as for the article apply. Supplements are not edited by *Eurosurveillance* and the journal is not responsible for the maintenance of any links or email addresses provided therein.

**07/09/2022**	KU727766.1	355	5166	6.87	10	27712580	Parvoviridae	Parus major densovirus	unclassified Parus major densovirus subspecies/strain	NA	NA	19	Sample
**07/09/2022**	AY629583.1	353	4682	7.54	20	27712580	Parvoviridae	Avian dependoparvovirus 1	Avian adeno-associated virus strain DA-1	NA	NA	19	Sample
**07/09/2022**	AB041962.1	574	2908	19.74	26	27712580	Anelloviridae	Torque teno mini virus 5	unclassified Torque teno mini virus 5 subspecies/strain	NA	NA	19	Sample
**07/09/2022**	JX262162.1	2764	4939	55.96	611	27712580	Polyomaviridae	Deltapolyomavirus decihominis	unclassified Deltapolyomavirus decihominis subspecies/strain	NA	NA	19	Sample
**07/09/2022**	MF588693.1	4516	7226	62.5	870	27712580	Papillomaviridae	Gammapapillomavirus sp.	unclassified Gammapapillomavirus sp. subspecies/strain	NA	NA	19	Sample
**07/09/2022**	AB017613.1	2350	3818	61.55	1242	27712580	Anelloviridae	Torque teno virus 16	unclassified Torque teno virus 16 subspecies/strain	NA	NA	19	Sample
**14/09/2022**	FJ445140.1	291	7136	4.08	10	14298396	Picornaviridae	Rhinovirus A	rhinovirus A56	32.7	NA	18	Sample
**14/09/2022**	AF536531.1	213	9812	2.17	11	14298396	Dicistroviridae	Aphid lethal paralysis virus	unclassified Aphid lethal paralysis virus subspecies/strain	NA	NA	18	Sample
**14/09/2022**	AB041962.1	210	2908	7.22	14	14298396	Anelloviridae	Torque teno mini virus 5	unclassified Torque teno mini virus 5 subspecies/strain	NA	NA	18	Sample
**14/09/2022**	KM085343.1	266	7278	3.65	20	14298396	Papillomaviridae	Gammapapillomavirus 24	Human papillomavirus 197	NA	NA	18	Sample
**14/09/2022**	U31779.1	486	7779	6.25	51	14298396	Papillomaviridae	Betapapillomavirus 1	human papillomavirus 21	NA	NA	18	Sample
**14/09/2022**	KU645789.1	595	10056	5.92	72	14298396	Iflaviridae	Moku virus	unclassified Moku virus subspecies/strain	NA	NA	18	Sample
**14/09/2022**	AB060597.1	749	3246	23.07	204	14298396	Anelloviridae	Torque teno virus 24	unclassified Torque teno virus 24 subspecies/strain	NA	NA	18	Sample
**14/09/2022**	KY040275.1	1518	188253	0.81	212	14298396	Poxviridae	Molluscum contagiosum virus	Molluscum contagiosum virus subtype 1	NA	NA	18	Sample
**14/09/2022**	FR751463.1	1450	3725	38.93	332	14298396	Anelloviridae	Torque teno virus	unclassified Torque teno virus subspecies/strain	NA	NA	18	Sample
**28/09/2022**	EF444549.1	329	5229	6.29	20	12518762	Polyomaviridae	Betapolyomavirus quartihominis	WU Polyomavirus	NA	NA	20	Sample
**28/09/2022**	AB060597.1	711	3246	21.9	117	12518762	Anelloviridae	Torque teno virus 24	unclassified Torque teno virus 24 subspecies/strain	NA	NA	20	Sample
**28/09/2022**	JX262162.1	2346	4939	47.5	201	12518762	Polyomaviridae	Deltapolyomavirus decihominis	unclassified Deltapolyomavirus decihominis subspecies/strain	NA	NA	20	Sample
**05/10/2022**	KU727766.1	394	5166	7.63	11	19405330	Parvoviridae	Parus major densovirus	unclassified Parus major densovirus subspecies/strain	NA	NA	NA	Sample
**12/10/2022**	AB028668.1	281	3787	7.42	33	19253612	Anelloviridae	Torque teno virus 15	unclassified Torque teno virus 15 subspecies/strain	NA	NA	20	Sample
**12/10/2022**	KY369878.1	1096	7052	15.54	90	19253612	Picornaviridae	Rhinovirus C	rhinovirus C43	30.3	NA	20	Sample

This supplementary material is hosted by Eurosurveillance as supporting information alongside the article "Shotgun metagenomics on indoor air for surveillance of respiratory, enteric, and skin viruses in a Belgian daycare setting, January to December 2022", on behalf of the authors, who remain responsible for the accuracy and appropriateness of the content. The same standards for ethics, copyright, attributions and permissions as for the article apply. Supplements are not edited by *Eurosurveillance* and the journal is not responsible for the maintenance of any links or email addresses provided therein.

**12/10/2022**	AF043303.1	3762	4679	80.4	1444	19253612	Parvoviridae	Adeno-associated dependoparvovirus A	adeno-associated virus 2	NA	NA	20	Sample
**19/10/2022**	KY316160.1	393	34169	1.15	27	20826006	Adenoviridae	Human mastadenovirus F	Human adenovirus 41	32.8	37.2	NA	Sample
**19/10/2022**	NC_007455.1	548	5299	10.34	44	20826006	Parvoviridae	Primate bocaparvovirus 1	unclassified Primate bocaparvovirus 1 subspecies/strain	31.2	NA	NA	Sample
**19/10/2022**	MF588728.1	994	7209	13.79	46	20826006	Papillomaviridae	Gammapapillomavirus 16	unclassified Gammapapillomavirus 16 subspecies/strain	NA	NA	NA	Sample
**19/10/2022**	KU727766.1	451	5166	8.73	46	20826006	Parvoviridae	Parus major densovirus	unclassified Parus major densovirus subspecies/strain	NA	NA	NA	Sample
**19/10/2022**	JX262162.1	758	4939	15.35	58	20826006	Polyomaviridae	Deltapolyomavirus decihominis	unclassified Deltapolyomavirus decihominis subspecies/strain	NA	NA	NA	Sample
**19/10/2022**	KY369878.1	1092	7052	15.48	143	20826006	Picornaviridae	Rhinovirus C	rhinovirus C43	32.1	NA	NA	Sample
**19/10/2022**	HM011556.1	5379	5387	99.85	4107	20826006	Polyomaviridae	Alphapolyomavirus quintihominis	Merkel cell polyomavirus	NA	NA	NA	Sample
**26/10/2022**	FN665693.1	113	1356	8.33	13	21012810	Sedoreoviridae	Rotavirus A		NA	*Not detected*	23	Sample
**26/10/2022**	KU356605.1	162	751	21.57	14	21012810	Sedoreoviridae	Rotavirus A		NA	*Not detected*	23	Sample
**26/10/2022**	JQ919995.1	152	2769	5.49	14	21012810	Sedoreoviridae	Rotavirus F		NA	NA	23	Sample
**26/10/2022**	X86560.1	212	7431	2.85	14	21012810	Caliciviridae	Sapporo virus	Sapporo virus-Manchester	NA	Not detected	23	Sample
**26/10/2022**	KP298674.1	400	7429	5.38	16	21012810	Caliciviridae	Sapporo virus	Sapovirus Hu/GI.1/Seoul/ROK62/2013/KOR	NA	Not detected	23	Sample
**26/10/2022**	JX262162.1	796	4939	16.12	102	21012810	Polyomaviridae	Deltapolyomavirus decihominis	unclassified Deltapolyomavirus decihominis subspecies/strain	NA	NA	23	Sample
**26/10/2022**	AF043303.1	1598	4679	34.15	280	21012810	Parvoviridae	Adeno-associated dependoparvovirus A	adeno-associated virus 2	NA	NA	23	Sample
**26/10/2022**	GU345044.1	952	5421	17.56	361	21012810	Polyomaviridae	Gammapolyomavirus secanaria	Canary polyomavirus	NA	NA	23	Sample
**26/10/2022**	NC_007455.1	1948	5299	36.76	394	21012810	Parvoviridae	Primate bocaparvovirus 1	unclassified Primate bocaparvovirus 1 subspecies/strain	29.5	NA	23	Sample
**26/10/2022**	EF444549.1	3494	5229	66.82	478	21012810	Polyomaviridae	Betapolyomavirus quartihominis	WU Polyomavirus	NA	NA	23	Sample
**09/11/2022**	NC_007455.1	403	5299	7.61	22	13856312	Parvoviridae	Primate bocaparvovirus 1	unclassified Primate bocaparvovirus 1 subspecies/strain	30.2	NA	NA	Sample
**09/11/2022**	KU727766.1	451	5166	8.73	36	13856312	Parvoviridae	Parus major densovirus	unclassified Parus major densovirus subspecies/strain	NA	NA	NA	Sample
**09/11/2022**	KY040275.1	1091	188253	0.58	40	13856312	Poxviridae	Molluscum contagiosum virus	Molluscum contagiosum virus subtype 1	NA	NA	NA	Sample

This supplementary material is hosted by Eurosurveillance as supporting information alongside the article "Shotgun metagenomics on indoor air for surveillance of respiratory, enteric, and skin viruses in a Belgian daycare setting, January to December 2022", on behalf of the authors, who remain responsible for the accuracy and appropriateness of the content. The same standards for ethics, copyright, attributions and permissions as for the article apply. Supplements are not edited by *Eurosurveillance* and the journal is not responsible for the maintenance of any links or email addresses provided therein.

**16/11/2022**	JX134046.1	218	2915	7.48	12	22190290	Anelloviridae	TTV-like mini virus	unclassified TTV-like mini virus subspecies/strain	NA	NA	NA	Sample
**16/11/2022**	KU727766.1	368	5166	7.12	19	22190290	Parvoviridae	Parus major densovirus	unclassified Parus major densovirus subspecies/strain	NA	NA	NA	Sample
**16/11/2022**	KY040275.1	1059	188253	0.56	24	22190290	Poxviridae	Molluscum contagiosum virus	Molluscum contagiosum virus subtype 1	NA	NA	NA	Sample
**16/11/2022**	KT779555.1	492	29887	1.65	41	22190290	Coronaviridae	Human coronavirus HKU1	unclassified Human coronavirus HKU1 subspecies/strain	NA	NA	NA	Sample
**16/11/2022**	KY629935.1	335	7130	4.7	75	22190290	Picornaviridae	Rhinovirus A	rhinovirus A59	32.9	NA	NA	Sample
**16/11/2022**	EF582387.1	1059	7086	14.94	129	22190290	Picornaviridae	Rhinovirus C	rhinovirus C6	32.9	NA	NA	Sample
**16/11/2022**	KY490071.1	1822	235397	0.77	147	22190290	Herpesviridae	Human betaherpesvirus 5	unclassified Human betaherpesvirus 5 subspecies/strain	32.8	NA	NA	Sample
**16/11/2022**	AF043303.1	2228	4679	47.62	257	22190290	Parvoviridae	Adeno-associated dependoparvovirus A	adeno-associated virus 2	NA	NA	NA	Sample
**16/11/2022**	NC_007455.1	2393	5299	45.16	322	22190290	Parvoviridae	Primate bocaparvovirus 1	unclassified Primate bocaparvovirus 1 subspecies/strain	32.4	NA	NA	Sample
**16/11/2022**	JX262162.1	3207	4939	64.93	481	22190290	Polyomaviridae	Deltapolyomavirus decihominis	unclassified Deltapolyomavirus decihominis subspecies/strain	NA	NA	NA	Sample
**23/11/2022**	AB041007.1	322	3852	8.36	13	14766256	Anelloviridae	Torque teno virus 1	unclassified Torque teno virus 1 subspecies/strain	NA	NA	NA	Sample
**23/11/2022**	AB038621.1	413	3676	11.24	16	14766256	Anelloviridae	Torque teno virus 29	unclassified Torque teno virus 29 subspecies/strain	NA	NA	NA	Sample
**23/11/2022**	JX262162.1	416	4939	8.42	18	14766256	Polyomaviridae	Deltapolyomavirus decihominis	unclassified Deltapolyomavirus decihominis subspecies/strain	NA	NA	NA	Sample
**23/11/2022**	AX025718.1	370	3313	11.17	19	14766256	Anelloviridae	Torque teno virus 18	unclassified Torque teno virus 18 subspecies/strain	NA	NA	NA	Sample
**23/11/2022**	KU727766.1	467	5166	9.04	28	14766256	Parvoviridae	Parus major densovirus	unclassified Parus major densovirus subspecies/strain	NA	NA	NA	Sample
**23/11/2022**	Y15174.1	1846	7549	24.45	264	14766256	Papillomaviridae	Betapapillomavirus 3	human papillomavirus 76	NA	NA	NA	Sample
**07/12/2022**	NC_007455.1	610	5299	11.51	15	11691674	Parvoviridae	Primate bocaparvovirus 1	unclassified Primate bocaparvovirus 1 subspecies/strain	30	NA	NA	Sample
**07/12/2022**	MG846442.1	280	4432	6.32	57	11691674	Parvoviridae	Galliform chaphamaparvovirus 2	Chicken chapparvovirus 2	NA	NA	NA	Sample
**07/12/2022**	KY490071.1	3064	235397	1.3	403	11691674	Herpesviridae	Human betaherpesvirus 5	unclassified Human betaherpesvirus 5 subspecies/strain	32.2	NA	NA	Sample
**NA**	U31779.1	233	7779	3	39	14703762	Papillomaviridae	Betapapillomavirus 1	human papillomavirus 21	NA	NA	NA	Control
