# Supplementary material for: Shotgun metagenomics on indoor air for surveillance of respiratory, enteric, and skin viruses in a Belgian daycare setting, January to December 2022
Source: Euro Surveill. 2025 Sep 25;30(38):2400711. doi: 10.2807/1560-7917.ES.2025.30.38.2400711 (PMC12475893; doi:10.2807/1560-7917.ES.2025.30.38.2400711)
Supplement: SupplementaryInformation2 [file 24-00711_SupplementaryInformation_2.pdf]

This supplementary material is hosted by Eurosurveillance as supporting information alongside the article " Shotgun metagenomics on indoor air for surveillance of respiratory, enteric, and skin viruses in a Belgian daycare setting, January to December 2022", on behalf of the authors, who remain responsible for the accuracy and appropriateness of the content. The same standards for ethics, copyright, attributions and permissions as for the article apply. Supplements are not edited by Eurosurveillance and the journal is not responsible for the maintenance of any links or email addresses provided therein.

Supplementary information 2. S1. Monthly average PCR CT values per pathogen. Pathogens detected less than 10 times were excluded, blue shade shows confidence interval and was only calculated when more than 6 samples were positive for that month.

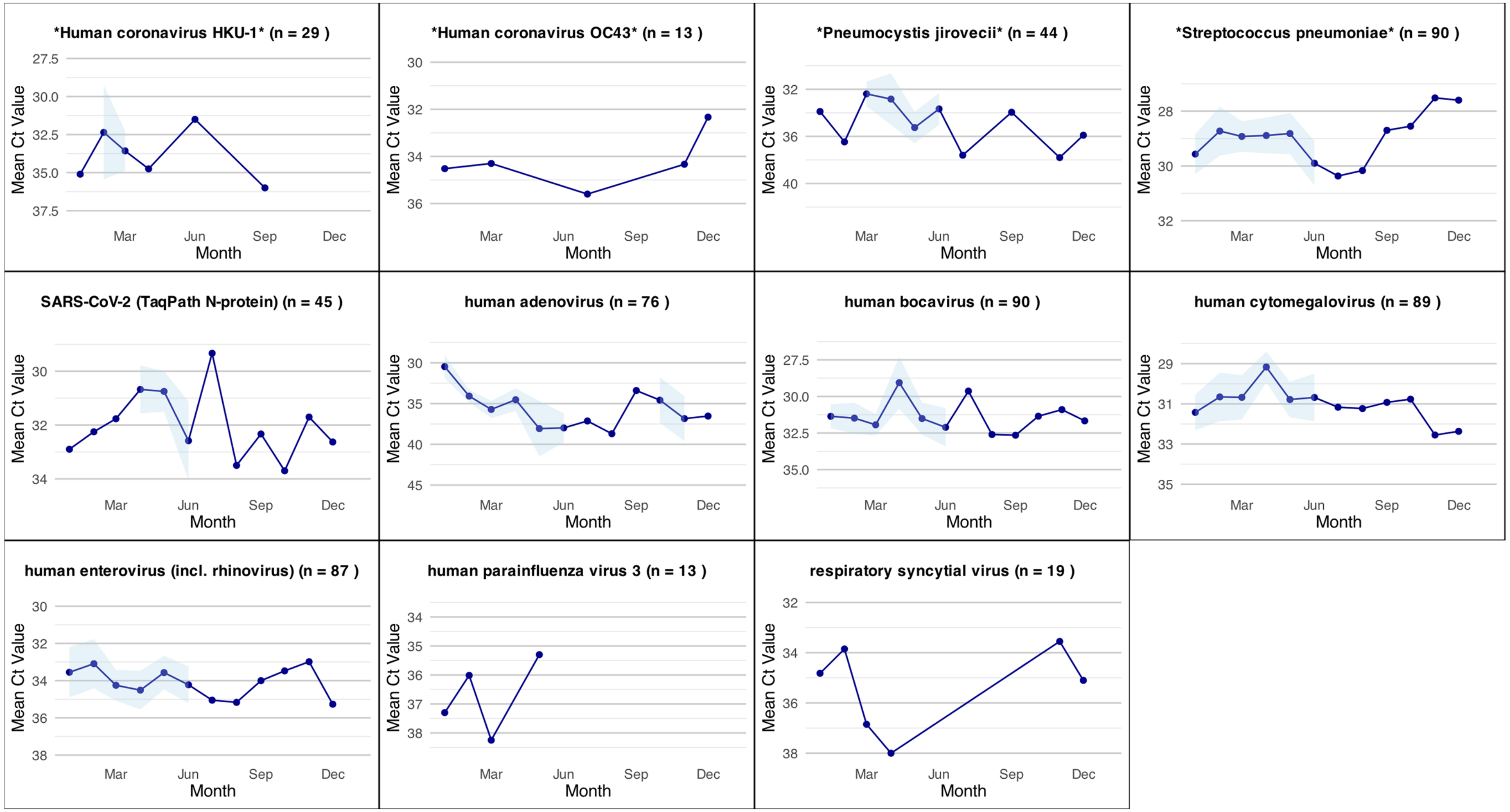

This supplementary material is hosted by Eurosurveillance as supporting information alongside the article " Shotgun metagenomics on indoor air for surveillance of respiratory, enteric, and skin viruses in a Belgian daycare setting, January to December 2022", on behalf of the authors, who remain responsible for the accuracy and appropriateness of the content. The same standards for ethics, copyright, attributions and permissions as for the article apply. Supplements are not edited by Eurosurveillance and the journal is not responsible for the maintenance of any links or email addresses provided therein.

Supplementary information 2.S2. Phylogenetic trees of a divergent canary polyomavirus, felis domesticus papillomavirus, Adeno-associated virus 2, human astrovirus, MW polyomavirus, WU polyomavirus and novel densovirus.

Canary polyomavirus

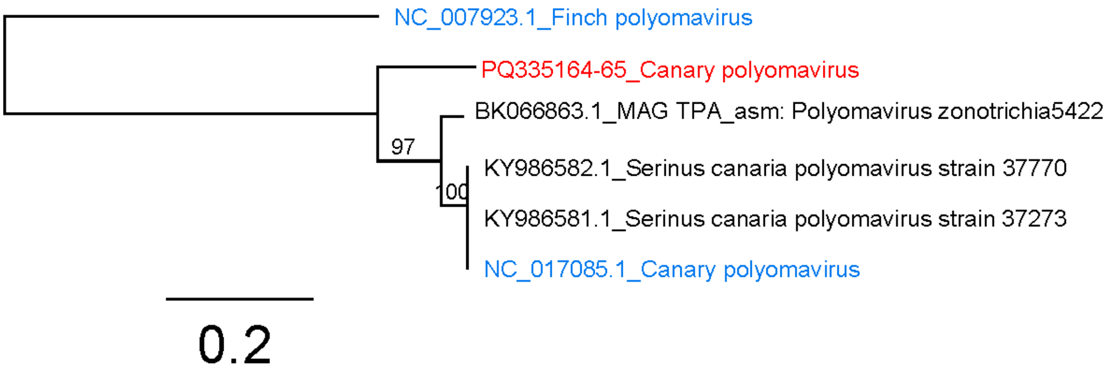

Felis domesticus papillomavirus

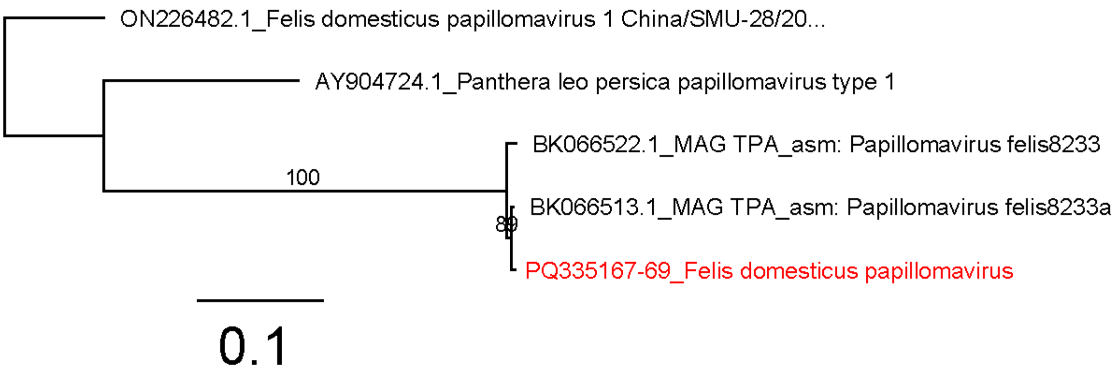

Adeno-associated virus 2

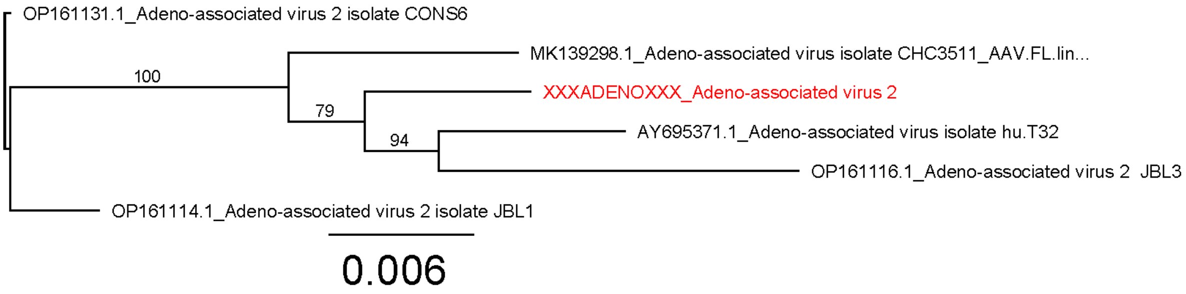

This supplementary material is hosted by Eurosurveillance as supporting information alongside the article " Shotgun metagenomics on indoor air for surveillance of respiratory, enteric, and skin viruses in a Belgian daycare setting, January to December 2022", on behalf of the authors, who remain responsible for the accuracy and appropriateness of the content. The same standards for ethics, copyright, attributions and permissions as for the article apply. Supplements are not edited by Eurosurveillance and the journal is not responsible for the maintenance of any links or email addresses provided therein.

Human astrovirus 4 (Mamastrovirus1)

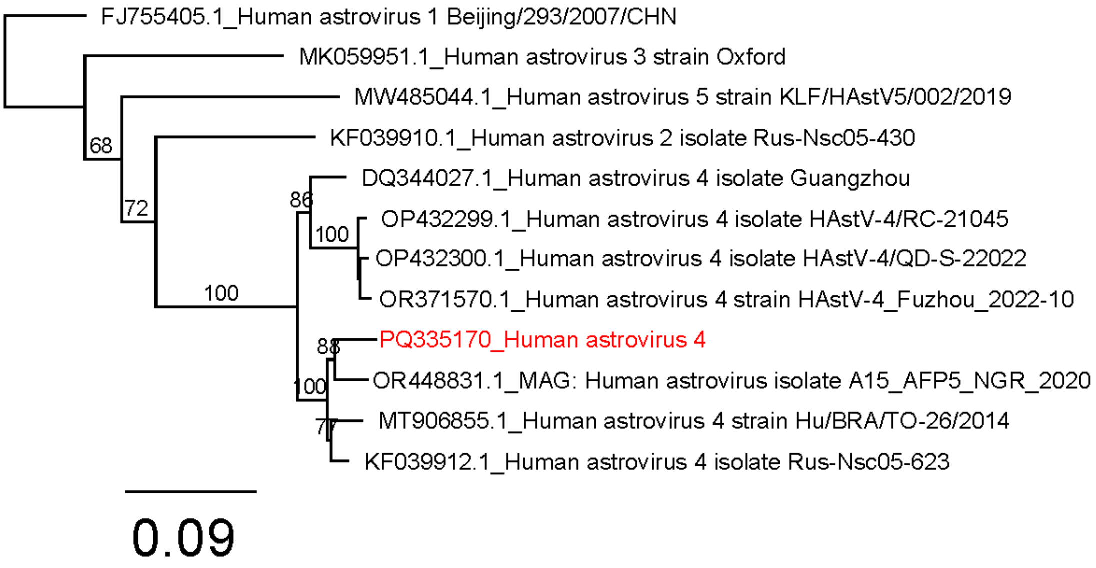

Human polyomavirus 10 (MW polyomavirus), Deltapolyomavirus decihominis

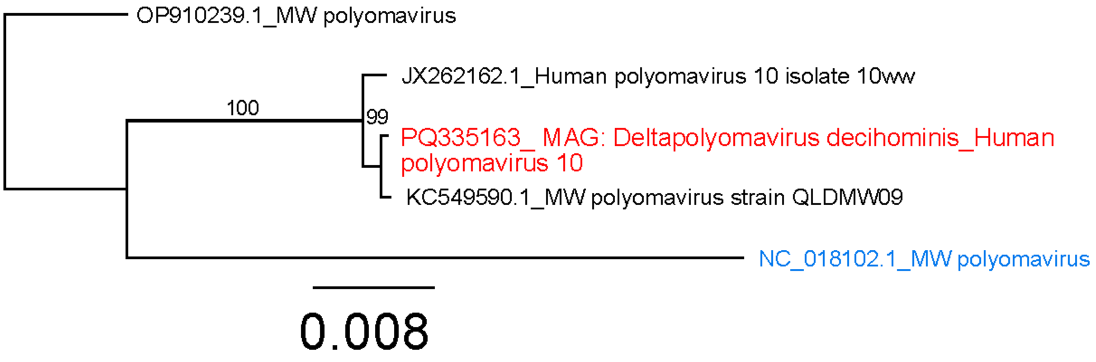

WU polyomavirus – Betapolyomavirus quartihominis

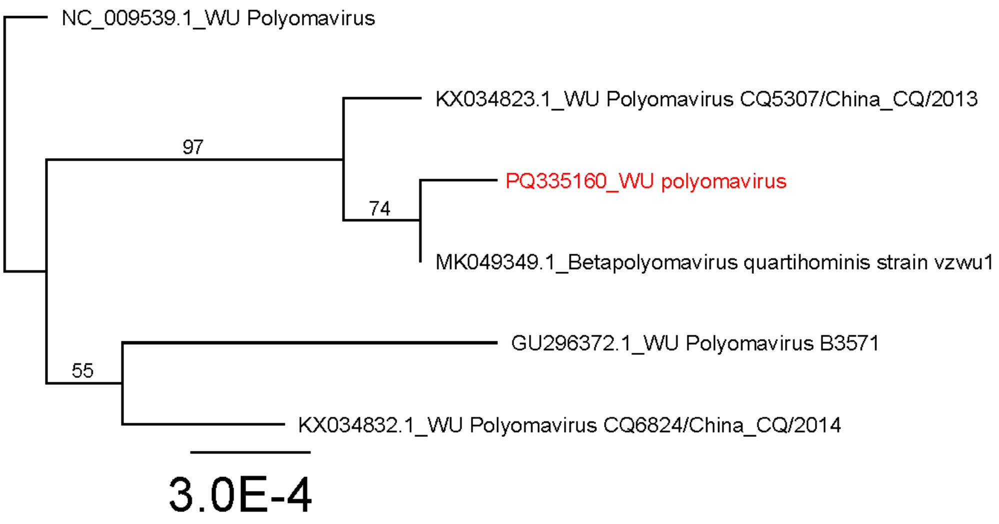

This supplementary material is hosted by Eurosurveillance as supporting information alongside the article " Shotgun metagenomics on indoor air for surveillance of respiratory, enteric, and skin viruses in a Belgian daycare setting, January to December 2022", on behalf of the authors, who remain responsible for the accuracy and appropriateness of the content. The same standards for ethics, copyright, attributions and permissions as for the article apply. Supplements are not edited by Eurosurveillance and the journal is not responsible for the maintenance of any links or email addresses provided therein.

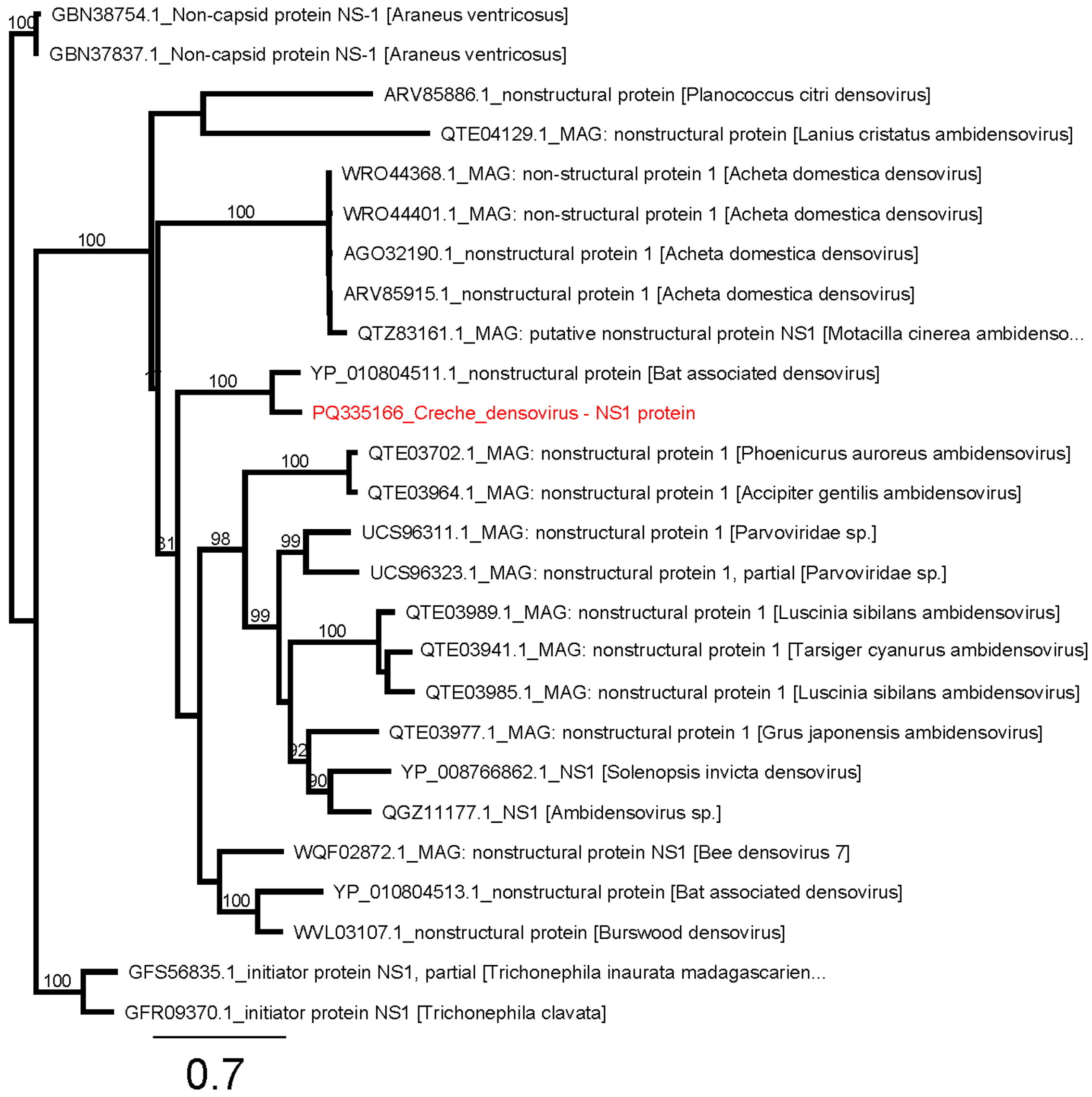

Red colored text shows the identified sequence from indoor air samples while blue-color sequences were recovered from the RefSeq database. All other sequences in black are sequences were sequences recovered from GenBank database. Phylogenetic trees were constructed using near-complete genomes (>95% horizontal coverage) for AAV-2 and human polyomaviruses. For human astrovirus 4, multiple contigs from ORF1a, ORF1b, and ORF2, comprising up to 71% of the genome (3.6 kbp), were combined to construct the phylogenetic tree. The phylogenetic tree for Canary polyomavirus was built using two contigs (1.9 kbp and 580 bp), covering both VP2 and VP1. The phylogenetic tree for Felis domesticus papillomavirus was constructed using a 4.4 kbp consensus sequence, corresponding to the E2 and L1 proteins. Phylogenetic tree of novel densovirus (*Creche densovirus*) was constructed using amino acid sequence of NS1 protein.

This supplementary material is hosted by Eurosurveillance as supporting information alongside the article " Shotgun metagenomics on indoor air for surveillance of respiratory, enteric, and skin viruses in a Belgian daycare setting, January to December 2022", on behalf of the authors, who remain responsible for the accuracy and appropriateness of the content. The same standards for ethics, copyright, attributions and permissions as for the article apply. Supplements are not edited by Eurosurveillance and the journal is not responsible for the maintenance of any links or email addresses provided therein.

Supplementary information 2. S3. WU Polyomavirus number of differences among (near) complete genomes.

A. Mutations compared to first two samples

Position \ Sample	30.05 and 15.06	22.06		06.07
	Nucleotide	Nucleotide	aa	
**583**	C	-> T	Syn	Y (C-85% or T-15%)
**1040**	A	-> C	N -> T	M (A-83% or C-17%)
**1251**	T	-> C	Syn	Y (C-16.7% or T-82.8%)
**1524**	A	-> G	Syn	R (A-70.7% or G-27.7%)
**2113**	C	-> T	P -> S	Y (C-80.2% or T-19.6%)
**2290**	T	-> C	Y -> H	Y (C-41.3% or T-58.5%)
**2979**	T	-> G	N -> K	K (T-52.1% or G-47.7%)
**4047**	G	-> A	Syn	R (G-61.7% or A-37.3%)
**4359**	G	-> C	L -> F	S (G-62.2% or C-37.2%)
**4668**	C	-> G	Syn	S (G-33.8% or C-65.8%)
**5211**	G	-> A	Syn	R (A-36.5% or G-63.5%)
**5216**	T	-> G	V -> G	K (G-37.1% or T-62.9%)

Below, you can see differences of consensus sequences from each sample (B) and close-up of two mutations at the end of the genome (C) .

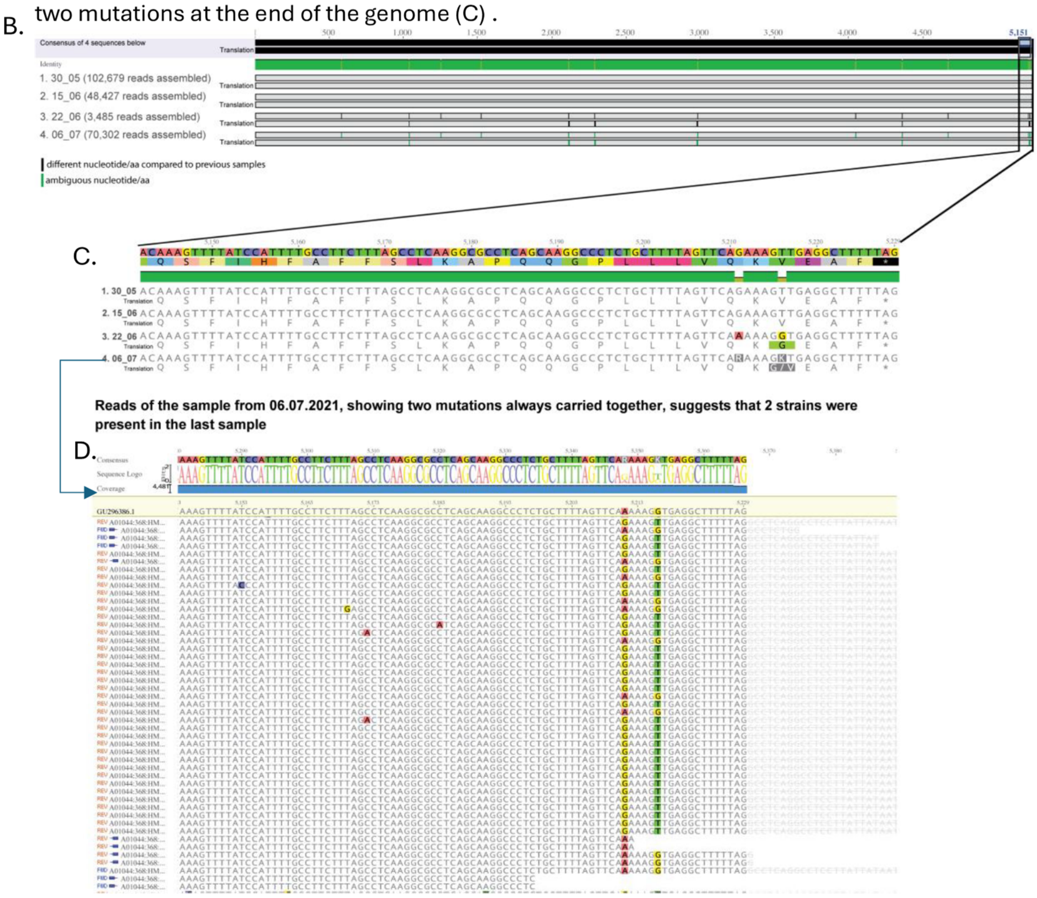

This supplementary material is hosted by Eurosurveillance as supporting information alongside the article " Shotgun metagenomics on indoor air for surveillance of respiratory, enteric, and skin viruses in a Belgian daycare setting, January to December 2022", on behalf of the authors, who remain responsible for the accuracy and appropriateness of the content. The same standards for ethics, copyright, attributions and permissions as for the article apply. Supplements are not edited by Eurosurveillance and the journal is not responsible for the maintenance of any links or email addresses provided therein.

Supplementary information 2. S4. Metadata.

	sampling_Date	humidity_mean	temperature_mean	co2_mean	co2_max	sampling_duration	Month	Windows	#people
**1**	**10/01/2022**	30.77	20.63	835.54	1000.00	2.25	January	Closed	15
**2**	**19/01/2022**	30.77	21.00	574.54	607.00	2.37	January	Closed	6
**3**	**24/01/2022**	29.10	20.40	594.90	717.00	1.88	January	Closed	10
**4**	**26/01/2022**	29.27	20.99	697.18	717.00	1.88	January	Closed	10
**5**	**31/01/2022**	34.82	20.31	829.27	917.00	1.95	January	Closed	15
**6**	**07/02/2022**	36.43	20.69	1009.00	1122.00	1.85	February	Closed	15
**7**	**21/02/2022**	32.55	20.46	827.64	1064.00	2.20	February	Closed	20
**8**	**23/02/2022**	34.23	21.29	709.77	868.00	2.08	February	Closed	16
**9**	**04/03/2022**	25.15	21.24	832.15	941.00	2.25	March	Closed	17
**10**	**07/03/2022**	24.64	21.69	1035.91	1119.00	2.03	March	Closed	23
**11**	**09/03/2022**	23.15	22.02	731.46	971.00	2.20	March	Closed	18
**12**	**11/03/2022**	24.43	21.86	711.21	776.00	2.25	March	Closed	23
**13**	**14/03/2022**	38.09	21.18	900.36	991.00	2.18	March	Closed	21
**14**	**16/03/2022**	34.09	21.83	820.73	894.00	1.95	March	Closed	20
**15**	**21/03/2022**	32.33	21.25	919.67	1165.00	2.25	March	Closed	22
**16**	**23/03/2022**	29.82	21.57	734.18	963.00	1.83	March	Open	18
**17**	**30/03/2022**	36.42	21.07	900.92	1161.00	2.42	March	Closed	17
**18**	**04/04/2022**	28.89	20.30	831.67	989.00	1.80	April	Closed	15
**19**	**08/04/2022**	35.45	20.81	910.09	1017.00	1.83	April	Closed	16
**20**	**25/04/2022**	38.70	21.35	925.00	985.00	2.05	April	Open	22
**21**	**06/05/2022**	NA	NA	NA	NA	NA	May	Open	20
**22**	**11/05/2022**	NA	NA	NA	NA	NA	May	Open	20
**23**	**18/05/2022**	NA	NA	NA	NA	NA	May	Open	16
**24**	**23/05/2022**	NA	NA	NA	NA	NA	May	Open	21
**25**	**30/05/2022**	40.20	21.22	730.30	826.00	2.13	May	Open	18
**26**	**08/06/2022**	NA	NA	NA	NA	NA	June	Open	18
**27**	**15/06/2022**	NA	NA	NA	NA	NA	June	Open	14
**28**	**22/06/2022**	NA	NA	NA	NA	NA	June	Open	19
**29**	**29/06/2022**	54.50	23.12	745.80	1030.00	1.92	June	Open	23
**30**	**06/07/2022**	47.94	22.48	779.72	1102.00	3.35	July	Open	18
**31**	**13/07/2022**	51.00	24.38	630.18	718.00	1.87	July	Open	18
**32**	**07/09/2022**	NA	NA	NA	NA	NA	September	Open	19
**33**	**14/09/2022**	58.17	23.19	636.17	866.00	2.02	September	Open	18
**34**	**28/09/2022**	NA	NA	NA	NA	NA	September	Open	20
**35**	**05/10/2022**	NA	NA	NA	NA	NA	October	Open	NA

This supplementary material is hosted by Eurosurveillance as supporting information alongside the article " Shotgun metagenomics on indoor air for surveillance of respiratory, enteric, and skin viruses in a Belgian daycare setting, January to December 2022", on behalf of the authors, who remain responsible for the accuracy and appropriateness of the content. The same standards for ethics, copyright, attributions and permissions as for the article apply. Supplements are not edited by Eurosurveillance and the journal is not responsible for the maintenance of any links or email addresses provided therein.

**36**	**12/10/2022**	37.17	21.24	744.42	856.00	2.17	October	Open	20
**37**	**19/10/2022**	48.09	21.51	802.45	906.00	1.92	October	Open	NA
**38**	**26/10/2022**	54.42	21.93	798.58	912.00	2.08	October	Open	23
**39**	**09/11/2022**	48.00	21.69	704.29	780.00	2.07	November	Closed	NA
**40**	**16/11/2022**	47.00	21.70	924.30	1073.00	2.07	November	Closed	NA
**41**	**23/11/2022**	NA	NA	NA	NA	NA	November	Closed	NA
**42**	**07/12/2022**	36.73	21.06	929.64	1027.00	2.12	December	Closed	NA

This supplementary material is hosted by Eurosurveillance as supporting information alongside the article " Shotgun metagenomics on indoor air for surveillance of respiratory, enteric, and skin viruses in a Belgian daycare setting, January to December 2022", on behalf of the authors, who remain responsible for the accuracy and appropriateness of the content. The same standards for ethics, copyright, attributions and permissions as for the article apply. Supplements are not edited by Eurosurveillance and the journal is not responsible for the maintenance of any links or email addresses provided therein.

Supplementary Information 2. S5. Non-human infecting viruses and relative abundance of viruses per sample.

A diverse array of plant and fungi infecting viruses were identified in 39/42 samples collected throughout the year. The identified viruses belonged to the families *Totiviridae* (predominantly fungi-infecting, n=27), *Tombusviridae* (plant-infecting, n=15), and 3 families of viruses known to infect both plants and fungi: *Genomoviridae* (n=17), *Partitiviridae* (n=9) and *Alphaflexiviridae* (n=18) (**Figure 4.B,** green panel and **Supplementary figure S5.2.A.**).

With respect to fungal viruses, we noted that some of the more abundantly identified viral genome sequences from the family *Totiviridae* were most closely related (~80% amino acid identity) to viruses known to infect fungi associated with humans: “Geotrichum candidum infecting totivirus” and “Malassezia-restricta infecting totivirus” (**Supplementary figure S5.2.A.**). Overall, totiviruses were consistently detected in February-March (10 out of 12 samples) and July-October (7 out of 9 samples) (**Supplementary figure S5.2.A.**). Although our NGS protocol is aimed at enriching for virus sequences, the *de novo* approach also identified contigs classified as *Malasseziaecea* and reads counts mapping to these contigs correlated strongly with the relative abundance of totiviruses (**Supplementary figure S5.2.A-B.**).

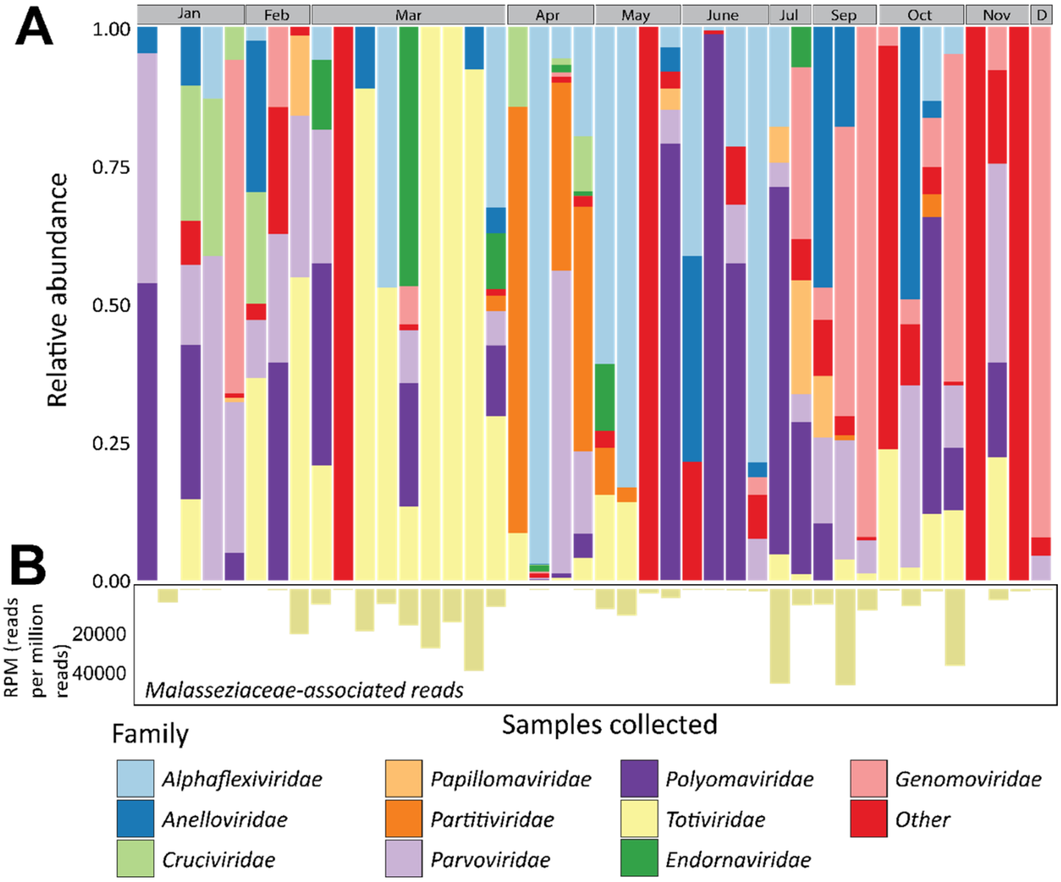

Supplementary figure S5.2.A. Relative abundance of different viral families detected in each sample. **B.** Reads mapping back to contigs of family *Malasseziaceae*, as RPM (reads per million).

This supplementary material is hosted by Eurosurveillance as supporting information alongside the article " Shotgun metagenomics on indoor air for surveillance of respiratory, enteric, and skin viruses in a Belgian daycare setting, January to December 2022", on behalf of the authors, who remain responsible for the accuracy and appropriateness of the content. The same standards for ethics, copyright, attributions and permissions as for the article apply. Supplements are not edited by Eurosurveillance and the journal is not responsible for the maintenance of any links or email addresses provided therein.

Supplementary Information 2. S6. Number of viral species identified using reference-guided assembly did not show any correlation with environmental parameters

We further explored correlations between environmental parameters and species identified per sample using reference-guided assembly results. No parameter correlated with species identified (p>0.05). It should be noted that we present these results for 42 samples and higher variation in species identified and mean CO2 values can be more informative and robust. On the other hand, known correlation between number of people present in the room and mean CO2 values have been confirmed in our study (p=0.02).

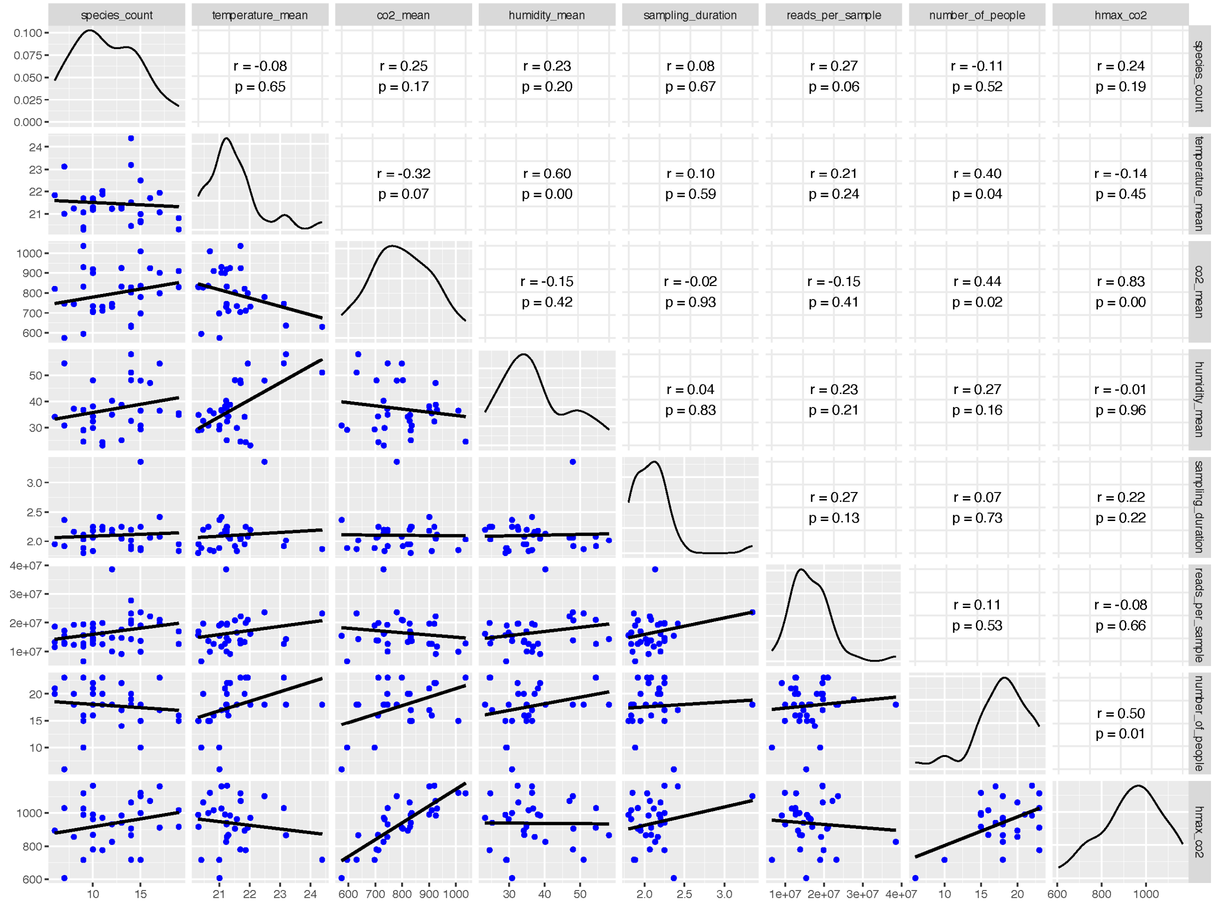

Supplementary Information 2. S6. Correlation coefficient of environmental parameters and species identified per sample.
